# Manipulating the Mitochondrial Genome To Enhance Cattle Embryo Development

**DOI:** 10.1534/g3.117.042655

**Published:** 2017-05-08

**Authors:** Kanokwan Srirattana, Justin C. St. John

**Affiliations:** * Centre for Genetic Diseases, Hudson Institute of Medical Research, Clayton, Victoria 3168, Australia; † Department of Molecular and Translational Science, Monash University, Clayton, Victoria 3168, Australia

**Keywords:** mitochondrial DNA, depletion, cattle, somatic cell nuclear transfer, embryo development

## Abstract

The mixing of mitochondrial DNA (mtDNA) from the donor cell and the recipient oocyte in embryos and offspring derived from somatic cell nuclear transfer (SCNT) compromises genetic integrity and affects embryo development. We set out to generate SCNT embryos that inherited their mtDNA from the recipient oocyte only, as is the case following natural conception. While SCNT blastocysts produced from Holstein (*Bos taurus*) fibroblasts were depleted of their mtDNA, and oocytes derived from Angus (*Bos taurus*) cattle possessed oocyte mtDNA only, the coexistence of donor cell and oocyte mtDNA resulted in blastocysts derived from nondepleted cells. Moreover, the use of the reprogramming agent, Trichostatin A (TSA), further improved the development of embryos derived from depleted cells. RNA-seq analysis highlighted 35 differentially expressed genes from the comparison between blastocysts generated from nondepleted cells and blastocysts from depleted cells, both in the presence of TSA. The only differences between these two sets of embryos were the presence of donor cell mtDNA, and a significantly higher mtDNA copy number for embryos derived from nondepleted cells. Furthermore, the use of TSA on embryos derived from depleted cells positively modulated the expression of *CLDN8*, *TMEM38A*, and *FREM1*, which affect embryonic development. In conclusion, SCNT embryos produced by mtDNA depleted donor cells have the same potential to develop to the blastocyst stage without the presumed damaging effect resulting from the mixture of donor and recipient mtDNA.

Assisted reproductive technologies, such as somatic cell nuclear transfer (SCNT), offer the potential to produce animals with enhanced genetic qualities by combining favorable chromosomal traits with favorable mitochondrial DNA (mtDNA) traits. During SCNT, a somatic donor cell is transferred into an enucleated recipient oocyte. Reconstructed oocytes are then activated chemically and cultured for 7 d to the blastocyst stage. The blastocyst is transferred to a recipient and an offspring is delivered (Wilmut *et al.* 1997). However, the overall efficiency of SCNT in domestic species is still low (reviewed by Keefer 2015), which is thought to be caused by incomplete epigenetic reprogramming (Jaenisch *et al.* 2002). For example, aberrant DNA methylation, histone methylation, and histone acetylation patterns have been identified at greater frequencies in SCNT cattle embryos when compared with *in vivo* or *in vitro* fertilized embryos (Bourc’his *et al.* 2001; Kang *et al.* 2002; Dean *et al.* 2003; Han *et al.* 2003; Santos *et al.* 2003). Consequently, Trichostatin A (TSA), a histone deacetylase inhibitor, has been widely used for enhancing SCNT efficiency in mouse (Kishigami *et al.* 2006; Rybouchkin *et al.* 2006), cattle (Akagi *et al.* 2011; Lee *et al.* 2011; Srirattana *et al.* 2012), pigs (Zhang *et al.* 2007; Li *et al.* 2008; Kim *et al.* 2011), rabbits (Shi *et al.* 2008), and sheep (Hu *et al.* 2012).

Nevertheless, there are still a number of confounding issues related to SCNT, especially the irregular patterns of transmission of the mitochondrial genome. MtDNA is a circular, double-stranded molecule that, in cattle, is ∼16.3 kb in size (Anderson *et al.* 1982). It encodes 13 protein-coding genes of the electron transfer chain that mediates the process of oxidative phosphorylation (Anderson *et al.* 1982), 22 tRNAs, and two rRNAs. It also has one noncoding region, the D-loop, which houses the control region, and two hypervariable regions.

MtDNA is normally maternally-only inherited (Birky 1995), and, as a result, a uniform population of mtDNA is transmitted through the female germ line from one generation to the next. In SCNT, as a whole donor cell is transferred into an enucleated oocyte, not only is the nuclear genome passed onto the oocyte, but also the mtDNA present in the mitochondria surrounding the donor cell (Takeda *et al.* 2003). Donor cells typically have between 0.5 and 8 × 10^2^ copies of mtDNA per cell (Burgstaller *et al.* 2007; Jiang *et al.* 2011), while cattle oocytes have between 3 and 6 × 10^5^ copies of mtDNA (May-Panloup *et al.* 2005; Iwata *et al.* 2011; Cree *et al.* 2015). As a result, two genetically diverse populations of mtDNA can coexist within the embryo, and, as mtDNA is prematurely replicated in SCNT-derived preimplantation embryos (Bowles *et al.* 2007), its presence is not just dependent on the amount of donor cell mtDNA that is introduced into the recipient oocyte (reviewed in St. John *et al.* 2010). Indeed, the transmission of donor cell mtDNA is a random event, with donor cell mtDNA contributing from 0 to 59% of the offspring’s total mtDNA content in livestock species (Steinborn *et al.* 2002; Hiendleder *et al.* 2003; Takeda *et al.* 2003). Furthermore, in mouse models, it is well documented that two genetically distinct populations of mtDNA can lead to severe metabolic syndromes and other disorders (Nakada *et al.* 2001; Acton *et al.* 2007). Many of these disorders are similar to those reported in SCNT animals (Cibelli *et al.* 2002). However, it is not only the mixing of donor cell and oocyte mtDNA that could lead to developmental abnormalities, but the donor cell could be contributing aged and damaged mitochondria to the oocyte, which could trigger processes such as apoptosis in the developing embryo (Li *et al.* 2000; Zhao *et al.* 2015), and lead to the occurrence of late-onset disease. Likewise, mitochondria isolated from somatic cells are metabolically more active (Takeda *et al.* 2010), which could be detrimental to early developmental outcomes.

One way to ensure that SCNT offspring inherit only oocyte mtDNA is to deplete the donor cell of its mtDNA prior to SCNT. This could be achieved by using DNA depletion agents such as ethidium bromide (Desjardins *et al.* 1985; Hayakawa *et al.* 1998; Lloyd *et al.* 2006; Lee *et al.* 2010) or 2′-3′ dideoxycytidine (ddC) (Brinkman and Kakuda 2000). They act by inhibiting the nuclear-encoded mtDNA-specific DNA Polymerase, DNA Polymerase Gamma (POLG), from interacting with the mitochondrial genome. Indeed, ddC is specific to POLG as it does not affect the polymerases that drive replication of nuclear DNA (*e.g.*, DNA polymerase alpha, delta, and epsilon; reviewed by Brinkman *et al.* 1998). Consequently, as mtDNA replication does not take place, the mtDNA content of the cell is diluted out with each cell division while in culture (Brinkman *et al.* 1999).

Here, we show that mtDNA-depleted cells can be used to generate viable blastocysts. When the reconstructed oocytes were cultured in the presence of TSA, they had increased development rates to the blastocyst stage. Furthermore, blastocysts derived from mtDNA-depleted cells were able to efficiently regulate their mtDNA copy number, and they exhibited different chromosomal gene expression profiles to blastocysts generated from nondepleted cells.

## Materials and Methods

All chemicals and reagents were purchased from Sigma-Aldrich (St. Louis, MO) unless otherwise specified.

### Donor cell preparation

Skin fibroblasts were obtained from an adult cow (Holstein No. 1, *Bos taurus*). Donor cell mtDNA was depleted from cattle fibroblasts by culturing the cells in Dulbecco’s Modified Eagle Medium (DMEM, Gibco, Grand Island, NY) supplemented with 10% fetal bovine serum (FBS, Gibco, batch number 1153562), in the presence of 20 µM 2′-3′-dideoxycytidine (ddC) and 50 µg/ml uridine at 37° under humidified atmosphere of 5% CO_2_ in air for 30 d (mtDNA depleted cell). To determine whether mtDNA copy number could recover following mtDNA depletion, depleted cells were continuously cultured in the absence of ddC for 14 d.

Prior to SCNT, mtDNA-depleted cells were harvested by standard trypsinization with TrypLE Express (Gibco), and resuspended in EMCARE holding solution (ICPbio Reproduction, Timru, Auckland, New Zealand). For nondepleted cells, fibroblast cells were synchronized to the G0/G1 phase by culture in DMEM supplemented with 0.5% FBS at 37° under humidified atmosphere of 5% CO_2_ in air for 2–3 d.

### SCNT embryo production

To evaluate the effects of depleting mtDNA from the donor cells and TSA supplementation on the developmental potential of SCNT embryos, four different groups of embryos were produced: (1) embryos derived from nondepleted cells in the absence of TSA; (2) embryos derived from nondepleted cells in the presence of TSA; (3) embryos derived from depleted cells in the absence of TSA; and (4) embryos derived from depleted cells in the presence of TSA.

Oocyte preparation and SCNT were performed as previously described (Srirattana *et al.* 2012). Briefly, cumulus oocyte complexes (COCs) were aspirated from follicles of 2–3 mm from abattoir-derived ovaries (Angus, *B. taurus*). Then, the COCs were cultured in IVM medium [TCM199 supplemented with 10% FBS, 50 IU/ml hCG (Chorulon, Intervet, Bendigo East, VIC, Australia), 0.5 µg/ml FSH (Folltropin-V, Bioniche Animal Health, Belleville, ON, Canada), 1 µg/ml 17β-estradiol] at 38.5° under humidified atmosphere of 5% CO_2_ in air for 19 hr. Cumulus cells were then removed from COCs by repeated pipetting in 0.2% hyaluronidase. Enucleation was performed in 5 µg/ml cytochalasin B by aspirating the spindle from metaphase II (MII) oocytes using a micropipette (OD 12 µm, ID 9 µm, The Pipette Company, Adelaide, SA, Australia) under the Oosight Imaging System (CRi, Woburn, MA). A single donor cell from mtDNA-depleted cells (passage number 11) or nondepleted cells (passage number 9) was then inserted into the perivitelline space of each enucleated oocyte. The donor cell–cytoplast couplets were fused in Zimmermann fusion media (Zimmermann and Vienken 1982) using two direct current pulses of 22 V and 14 µs generated by a BTX Electro Cell Manipulator 200 (BTX, San Diego, CA).

Reconstructed oocytes were separated into two groups (absence of TSA, and presence of TSA). For the absence of TSA group, the reconstructed oocytes were placed in EMCARE holding solution for 1 hr, and, then, the success of the fusion was determined. The successfully fused oocytes were activated using 7% ethanol for 5 min at room temperature followed by 1.25 µg/ml cytochalasin D, and 10 µg/ml cycloheximide at 38.5° under humidified atmosphere of 5% CO_2_, 5% O_2_ and 90% N_2_ for 5 hr. After activation, the embryos were cultured in CR1aa medium (Rosenkrans *et al.* 1993) at 38.5° under humidified atmosphere of 5% CO_2_, 5% O_2_ and 90% N_2_ for 7 d. A half volume of CR1aa medium was replaced at days 3 and 5 of culture. In the presence of TSA, just minutes after fusion, reconstructed oocytes were placed in EMCARE holding solution supplemented with 50 nM TSA for 1 hr. Successfully fused oocytes were activated and cultured in medium supplemented with TSA for up to 10 hr (Srirattana *et al.* 2012). After that, embryos were continuously cultured in the medium without TSA for 7 d until they reached the blastocyst stage. A half volume of CR1aa medium was replaced at days 3 and 5 of culture. All experiments were run in parallel.

### Assessment of cell number of SCNT embryos

After 7 d of culture, SCNT embryos at the blastocyst stage (three individual embryos per group) were fixed with 4% paraformaldehyde, permeabilized in 0.1% Triton X-100, and then stained with 10 µl/ml of Hoechst 33258. The stained blastocysts were visualized using an inverted confocal microscope (FV1200, Olympus, Tokyo, Japan). The number of cells were determined by counting the number of Hoechst stained nuclei.

### MtDNA copy number analysis

Total DNA was extracted from mtDNA-depleted cells at various stages of depletion and recovery using the ISOLATE II Genomic DNA Kit (Bioline, Alexandria, NSW, Australia), and from oocytes at the MII stage and embryos at the blastocyst stage (10 individual oocytes/embryos per group) using the QIAamp DNA Micro Kit (Qiagen, Hilden, Germany), according to the manufacturer’s instructions.

Primers for assessing mtDNA copy number were designed from the cytochrome B (*CYTB*) region and the NADH dehydrogenase subunit 2 (*ND2*) region. For depleted cells, diploid nuclear genome analysis was also performed using the primers designed from the *POU* domain, class 5 transcription factor 1 (*POU*) region. Primer sequences are listed in Supplemental Material, Table S1 in File S4.

The mitochondrial and nuclear DNA target sequences were quantified by real-time polymerase chain reaction (PCR) using a Rotorgene 3000 real-time machine (Corbett Life Science, Sydney, NSW, Australia). The quantification of mtDNA was performed in 20 µl volumes of PCR mixtures containing 1× SensiMix SYBR Hi-ROX (Bioline), 0.25 µM of each primer, as listed in Table S1, and 20 ng of depleted cell DNA or 2 µl of embryo DNA. PCR amplification was carried out at 95° for 15 min, followed by 45 cycles of 95° for 15 sec, 58° (for *CYTB*) or 55° (for *ND2*) or 59° (for *POU*) for 15 sec, and 72° for 15 sec. The final elongation step was performed at 72° for 15 sec. The fluorescence signal was obtained via the FAM/Sybr channel at the elongation phase. From the first fluorescence signals, melting curve data were generated by heating PCR products from 47–98°, holding for 4 sec at each step. These data were used to determine the second extension phase, which was set at the temperature just before the start of the melt curve phase to exclude the negative effects of primer-dimerization. The second extension phase was set at 78° for *CYTB*, or 74° for *ND2*, and 80° (for *POU*) for 15 sec, and the second fluorescence signals were acquired.

To generate standards for real-time PCR, the *CYTB*, *ND2*, and *POU* regions were amplified from total DNA obtained from nondepleted cells. The PCR products were purified using the Isolate II PCR Gel Kit (Bioline), according to the manufacturer’s instructions, and were used as standards. For each run, a standard curve was generated from 10-fold serial dilutions (10^−1^ –10^−8^). mtDNA copy number per cell was calculated from the number of *CYTB* and *ND2* copies, and each divided by half the number of *POU* copies. Each sample was analyzed in triplicate to obtain the mean number of mtDNA copies.

### Analysis of the persistence of donor cell mtDNA in SCNT embryos

To determine the presence of donor cell mtDNA in SCNT embryos and offspring, single-nucleotide polymorphisms were analyzed using High Resolution Melt (HRM). The *CYTB* regions of the donor cells (Holstein) and recipient oocytes (Angus) were amplified and sequenced. The *CYTB* sequences were aligned using Multiple Sequence Alignment (Clustal Omega, EMBL-EBI, http://www.ebi.ac.uk/Tools/msa/clustalo/). HRM primers were designed from the *CYTB* region to detect single-nucleotide polymorphisms in donor cell and oocyte mtDNA. To generate standards for HRM, the purified *CYTB* PCR products were ligated into the pCR2.1 vector (Invitrogen, Carlsbad, CA). The ligated product was transformed into chemically competent cells (Max Efficiency DH5α, Invitrogen) by standard heat-shock transformation. Positive clones were selected by blue-white selection on LB agar plates containing 100 μg/ml ampicillin and 40 mg/ml 5-bromo-4-chloro-3-indolyl-β-d-galactopyranoside (X-gal). White colonies were cultured in LB broth containing 100 μg/ml ampicillin at 37°, 200 rpm for 16 hr. Plasmid preparation was performed using the ISOLATE II Plasmid Mini Kit (Bioline), according to the manufacturer’s instructions. To confirm the colonies were positive, the plasmids were amplified by PCR using M13F and M13R primers. The purified PCR products were sequenced by Capillary Electrophoresis using the ABI PRISM BigDye Terminator Cycle Sequencing Ready Reaction Kit (Applied Biosystems, Foster City, CA) on an ABI 3130xl Genetic Analyzer (Applied Biosystems, Hitachi, Tokyo, Japan) by the Monash Health Translation Precinct Biomedical Genomics Facility (Clayton, VIC, Australia). The plasmid mixtures of donor cell and recipient oocyte were prepared to the ratios of 100:0, 50:50, 30:70, 10:90, 3:97, 1:99, and 0:100. Total DNA was extracted from embryos at the blastocyst stage (five individual embryos per group) using the QIAamp DNA Micro Kit, according to the manufacturer’s instructions. The *CYTB* region was amplified by PCR. Ten microliter reaction mixtures consisted of 10 ng DNA sample or 10 ng plasmid mixture, 1× HRM master mix (TrendBio, Melbourne, VIC, Australia), 0.25 µM of each primer, as listed in Table S1. The amplification conditions were as follows: 95° for 2 min followed by 45 cycles of 94° for 30 sec and 61° for 30 sec. The final step was 94° for 30 sec. Each standard and sample were analyzed in triplicate. The single-nucleotide polymorphism in the PCR reaction was analyzed by the LightScanner system (Idaho Technology Inc., Salt Lake City, UT).

### RNA sequencing and analysis

RNA extraction from single blastocysts (three individual embryos per group) was performed using the Arcturus PicoPure RNA Isolation Kit (Life Technologies), according to the manufacturer’s instructions. Library preparations for RNA sequencing were performed using the Ovation RNA-Seq System V2 (NuGen, San Carlos, CA), according to the manufacturer’s instructions. First strand cDNA synthesis was performed using a DNA/RNA chimeric primer with random and polyT sequences. Second strand cDNA was synthesized by DNA polymerase, which primed at fragmentation of the RNA within the DNA/RNA complex. After that, the double-stranded cDNA was amplified by single primer isothermal amplification. Libraries were quantified using a Qubit fluorometer and an Agilent 2100 Bioanalyzer (Agilent Technology, Santa Clara, CA), and quantitative real time PCR. The denaturated cDNA (12 pM) was used for c-bot hybridization followed by on-board cluster generation in two lanes of an Illumina HiSequation 1500 Rapid with 50 bp read length, according to the manufacturer’s instructions (Illumina, San Diego, CA) by the Monash Health Translation Precinct (MHTP) Biomedical Genomics Facility. Raw reads from the libraries were quality checked using the FastQC tool (http://www.bioinformatics.babraham.ac.uk/projects/fastqc/). Sequence reads were assembled and mapped to the bovine reference genome (*B. taurus*, bosTau7) using the STAR software (Dobin *et al.* 2013). The number of reads mapped to each gene was calculated using the featureCounts tool (Liao *et al.* 2014). Those raw read counts were used for differential expression analysis. edgeR (Robinson *et al.* 2010) was used to analyze the differential expression of genes by pairwise comparisons between treatment groups. Multiple testing correction was performed in edgeR (empirical Bayes estimation) by applying the Benjamini and Hochberg method (Benjamini and Hochberg 1995) to *P*-values, to control for false-discovery rate (FDR). Differentially expressed genes with an FDR <0.05 were considered as significantly different (Driver *et al.* 2012; Schurch *et al.* 2016). Pathway analysis was performed using Ingenuity Pathway Analysis (IPA, QIAGEN Redwood City, CA, www.qiagenbioinformatics.com/products/ingenuity-pathway-analysis/), as described in Cagnone *et al.* (2016).

### Gene expression analysis

RNA extraction from single blastocysts (three individual embryos per group) was performed using the Arcturus PicoPure RNA Isolation Kit. cDNA synthesis was undertaken using the qScript Flex cDNA Kit (Quanta Biosciences, Gaithersburg, MD), according to the manufacturer’s instructions. Quantitative real-time PCR was performed using primer sets shown in Table S1 on a Rotorgene 3000 real-time machine with SensiMix SYBR Hi-ROX with the following thermal cycling conditions: 95° for 15 min, followed by 45 cycles of 95° for 15 sec, 55° (for *DNMT1* and *HDAC*) or 56° (for *NANOG*, *SOX2*, *CDX2*, *DNMT3a*, and *DNMT3b*), or 58° (for *ACTB*, *OCT4*, *TFAM*, and *POLGA*) for 15 sec and 72° for 15 sec. The final elongation step was performed at 72° for 15 sec. The second extension phase was set at 75° (for *TFAM*) or 78° (for *ACTB* and *POLGA*) or 80° (for *HDAC*, *OCT4*, *NANOG*, *DNMT1*, and *DNMT3b*) or 85° (for *SOX2*, *CDX2*, and *DNMT3a*) for 15 sec, and the second fluorescence signals were acquired. The relative quantification of gene expression was performed using the method described previously (Livak and Schmittgen 2001). *ACTB* was used as an internal standard for the analysis of relative transcript levels of each gene.

### Comparative genomic hybridization microarray

To determine the integrity of the genomes of depleted cells, comparative genomic hybridization (CGH) microarray was performed using the SurePrint G3 Bovine Genome CGH Microarray Kit (Agilent Technologies). DNA digestion, labeling, and hybridization were performed according to the manufacturer’s instructions. Briefly, DNA was extracted from depleted (*n* = 3) and nondepleted (*n* = 3) cells using the ISOLATE II Genomic DNA Kit, as previously described. DNA concentration and quality were measured using a NanoDrop ND-1000 Spectrophotometer (Thermo Scientific, Wilmington, DE); 500 ng of genomic DNA from depleted and nondepleted cells were digested using the SureTag DNA Labeling kit (Agilent Technologies), and were fluorescently labeled with Cy5 (for depleted cells) and Cy3 (for nondepleted cells, the reference). Following purification with the SureTag Purification Columns Kit (Agilent Technologies), the labeled samples were hybridized together at 65° on the microarray for 24 hr in a rotating Agilent hybridization oven at 20 rpm. The microarray was washed and scanned immediately at 3 µm resolution on a DNA microarray scanner (Agilent Technologies). Data were extracted from the scanned image using with Feature Extraction Software 11.0.1.1 (Agilent Technologies). Microarray data were analyzed using the Agilent Genomic Workbench version 7.0 software (Agilent Technologies), according to the manufacturer’s instructions. Aberration analysis was performed using the Aberration Detection Method 2 (ADM-2) algorithm, with a threshold of six and fuzzy zero turned on. Copy number variation was defined as absolute log2 ratio for duplication or deletion ≥0.25 and minimum number of probes = 3 (please refer to File S1).

### Statistical analysis

Statistical analysis of mtDNA copy number data was evaluated by one-way ANOVA with Tukey’s multiple comparisons test using GraphPad Prism version 6.01 (GraphPad Software Inc., La Jolla, CA). Data for preimplantation development, cell number, and gene expression were analyzed by ANOVA, and means were compared using Duncan’s Multiple Range Test using Statistical Analysis System version 9 (SAS Inst. Inc., Cary, NC). The number of differentially expressed genes was analyzed using edgeR (Robinson *et al.* 2010) at FDR <0.05.

### Data availability

RNA-sequencing data for SCNT embryos are deposited at the Sequence Read Archive, with SRA accession number: SRP078663. CGH microarray data of mtDNA depleted and nondepleted cells are deposited at the Gene Expression Omnibus with GEO accession number: GSE97912.

## Results

### MtDNA depletion of donor cells

**Figure 1:**
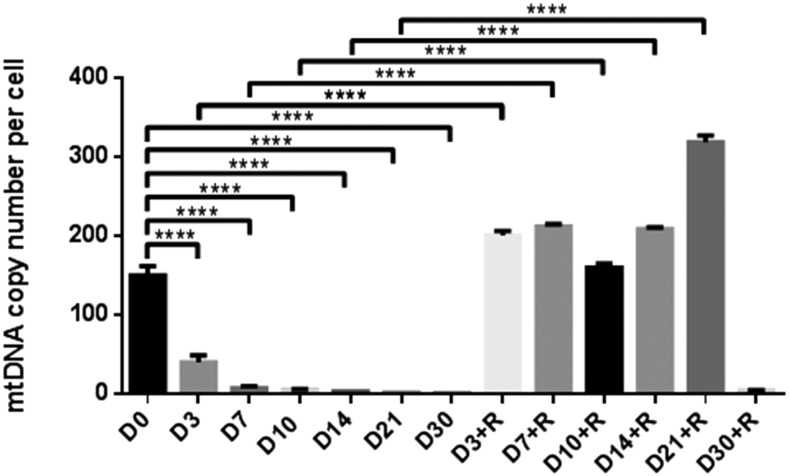
MtDNA copy number in cells following depletion for 3, 7, 10, 14, 21, and 30 d (D) with ddC, and a recovery period of 14 d (+R). Mean values (±SEM) are shown. The differences were analyzed for significance by one-way ANOVA with Tukey’s multiple comparisons test using GraphPad Prism version 6.01. ****P < 0.0001.

As the presence of donor cell and recipient oocyte mtDNA can be detrimental to development, we depleted fibroblasts derived from a *B. taurus* Holstein cow to be used as donor cells for SCNT. We depleted these cells over 30 d using the mtDNA depletion agent, ddC (20 µM) to monitor rates of depletion. There were significant decreases in mtDNA copy number from day 0 (150.11 ± 12.21, mean ± SEM) to day 30 (0.85 ± 0.02) of treatment (*P* < 0.0001, Figure 1), which resulted in <1 copy of mtDNA being present per cell. When cells depleted for 3, 7, 10, 14, 21, and 30 d were allowed to recover their mtDNA over 14 d in the absence of ddC, there were no significant differences in mtDNA copy number between cells depleted for 30 d, and those allowed to recover for 14 d in the absence of ddC (4.67 ± 0.19) indicating that they had lost their potential to replenish their mtDNA content. However, cells depleted for shorter periods of time replenished their mtDNA and exhibited significant differences compared to their respective depleted levels. Consequently, as cells depleted for 30 d do not have the capacity to replenish their own mtDNA, these cells were used as mtDNA depleted donor cells for SCNT.

We also determined the effects of mtDNA depletion on chromosome stability of the donor cells by using a CGH microarray. The results showed that there were no duplications or deletions for the chromosomes of mtDNA depleted cells when compared with nondepleted cells. This finding revealed that depleting mtDNA through the use of ddC did not have an adverse effect on chromosome stability in the donor cells.

### SCNT embryo production

To determine if depleted cells would be suitable candidates for SCNT, depleted and nondepleted donor cells were fused to MII oocytes. While the fusion rates (76.5%) were significantly lower than for nondepleted cells (93.7%, *P* < 0.05; Table 1), there was no significant difference in cleavage rates for embryos derived from depleted (78.7%) and nondepleted (87.1%) cells. However, the eight-cell, morula, and blastocyst rates for embryos derived from depleted cells (44.3, 20.7, and 20.1%, respectively) were significantly lower than those from nondepleted cells (61.3, 36.7, and 35.9%, respectively), which suggests that they were unable to overcome the developmental block associated with the activation of the embryonic genome (Memili *et al.* 1998).

**Table 1:** Developmental potential of cattle embryos derived from mtDNA depleted and nondepleted Holstein donor cells, and *B. taurus* recipient oocytes in the presence and absence of TSA

Donor Cell	TSA	Fused (%)	No. Embryos	Cleaved (%)*^a^*	No. (%) Embryo Developed to*^a^*		
					8-Cell	Morulae	Blastocysts
Nondepleted	−	254/271 (93.7 ± 1.6) b	248	216 (87.1 ± 0.3) b,c	152 (61.3 ± 2.2) b	91 (36.7 ± 2.9) b	89 (35.9 ± 3.4) b
	+	201/216 (93.1 ± 1.2) b	193	177 (91.7 ± 3.2) b	112 (58.0 ± 4.8) b,c	72 (37.3 ± 1.0) b	72 (37.3 ± 1.0) b
Depleted	−	189/247 (76.5 ± 1.7) c	174	137 (78.7 ± 5.0) c	77 (44.3 ± 4.6) c	36 (20.7 ± 3.4) c	35 (20.1 ± 3.8) c
	+	173/230 (75.2 ± 3.1) c	163	144 (88.3 ± 3.6) b,c	98 (60.1 ± 6.6) b	58 (35.6 ± 5.0) b	57 (35.0 ± 4.6) b

Values are mean ± SEM. Four replicates. All experiments were run in parallel. Different lower case letters (b and c) within a column indicate significant differences (*P* < 0.05, ANOVA with Duncan’s multiple range test using SAS version 9). −, absence of TSA; +, presence of TSA.

a Percentages calculated from the number of embryos that cleaved and developed to each stage.

To determine whether assisting the process of reprogramming would enhance development of embryos derived from depleted and nondepleted cells, we cultured reconstructed oocytes in the presence of TSA for 10 hr. The fusion and cleavage rates for embryos derived from depleted cells cultured in the presence of TSA (75.2 and 88.3%, respectively) were not significantly different when compared to those of embryos cultured in the absence of TSA (76.5 and 78.7%, respectively). Importantly, the presence of TSA promoted development through the developmental block associated with activation of the embryonic genome in SCNT embryos. Consequently, the eight-cell, morula, and blastocyst rates for embryos derived from depleted cells cultured in the presence of TSA (60.1, 35.6, and 35.0%, respectively) were significantly increased when compared to those of embryos cultured in the absence of TSA (44.3, 20.7, and 20.1%, respectively, *P* < 0.05). Furthermore, the morula and blastocyst rates matched those of embryos derived from nondepleted cells cultured in the presence and absence of TSA. Consequently, TSA enhanced development rates for embryos derived from depleted cells to levels equivalent to that of nondepleted cells.

**Figure 2:**
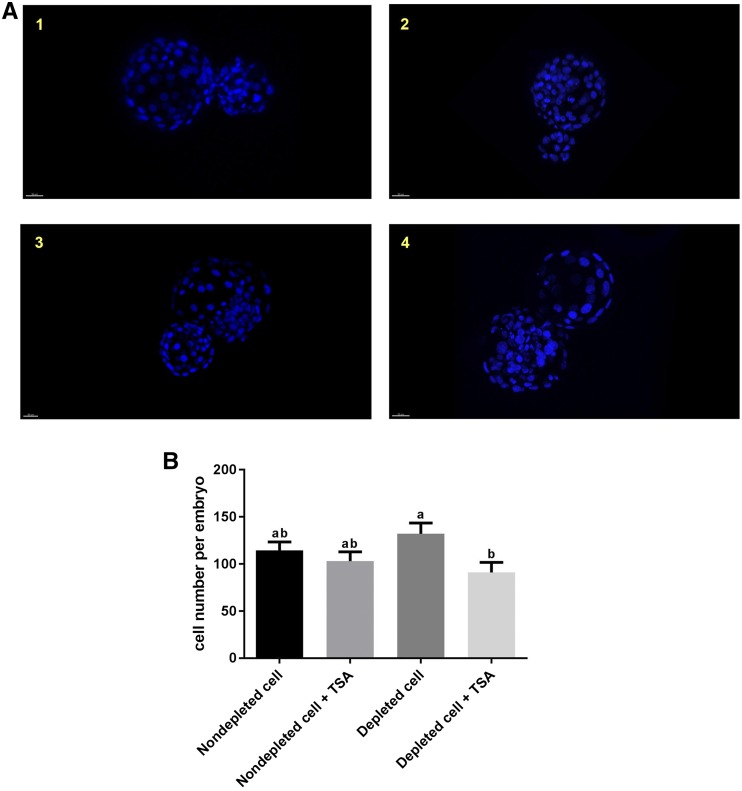
Assessment of SCNT embryos. (A) Fluorescence images of SCNT embryos at the blastocyst stage stained with Hoechst 33342: (1) embryos derived from nondepleted cells in the absence of TSA; (2) embryos derived from nondepleted cells in the presence of TSA; (3) embryos derived from depleted cells in the absence of TSA; and (4) embryos derived from depleted cells in the presence of TSA. (B) Cell number in SCNT embryos at the blastocyst stage (*n* = 3/group). Mean values (±SEM) are shown. Different superscripts within a column indicate significant differences (*P* < 0.05, ANOVA with Duncan’s multiple range test).

To determine if the cell number of SCNT embryos was affected by each treatment, we analyzed blastocysts at day 7 of culture following staining with 10 µg/ml Hoechst 33342 (Figure 2A). There were no significant differences in cell number for blastocysts derived from nondepleted (114 ± 9, mean ± SEM) and depleted cells (132 ± 12, Figure 2B). Although the presence of TSA increased blastocyst formation rates for embryos derived from depleted cells, these blastocysts had significantly fewer cells (91 ± 11, *P* < 0.05) when compared to blastocysts generated from depleted cells cultured in the absence of TSA (132 ± 12). However, TSA had no significant effect on cell number for blastocysts derived from nondepleted cells (presence of TSA, 103 ± 10; absence of TSA, 114 ± 9; Figure 2B).

Moreover, we performed a small-scale SCNT study using two different donor cell lines generated from two other adult Holstein cows (Holstein Nos. 2 and 3) derived from skin fibroblasts depleted of mtDNA, as described for Holstein No. 1. SCNT embryos were produced using these depleted cells cultured in the presence of TSA, as described for Holstein No. 1. Embryos produced from these cell lines also had the propensity to develop to the blastocyst stage (Table S2 in File S4), although their blastocyst rates (12–15%) were lower when compared with those of Holstein No. 1 (35%). However, as this was a smaller scale study, for consistency purposes, only SCNT embryos derived from the donor cells of Holstein No.1 were used for further analysis.

### Analysis of the persistence of donor cell mtDNA in SCNT embryos

**Figure 3:**
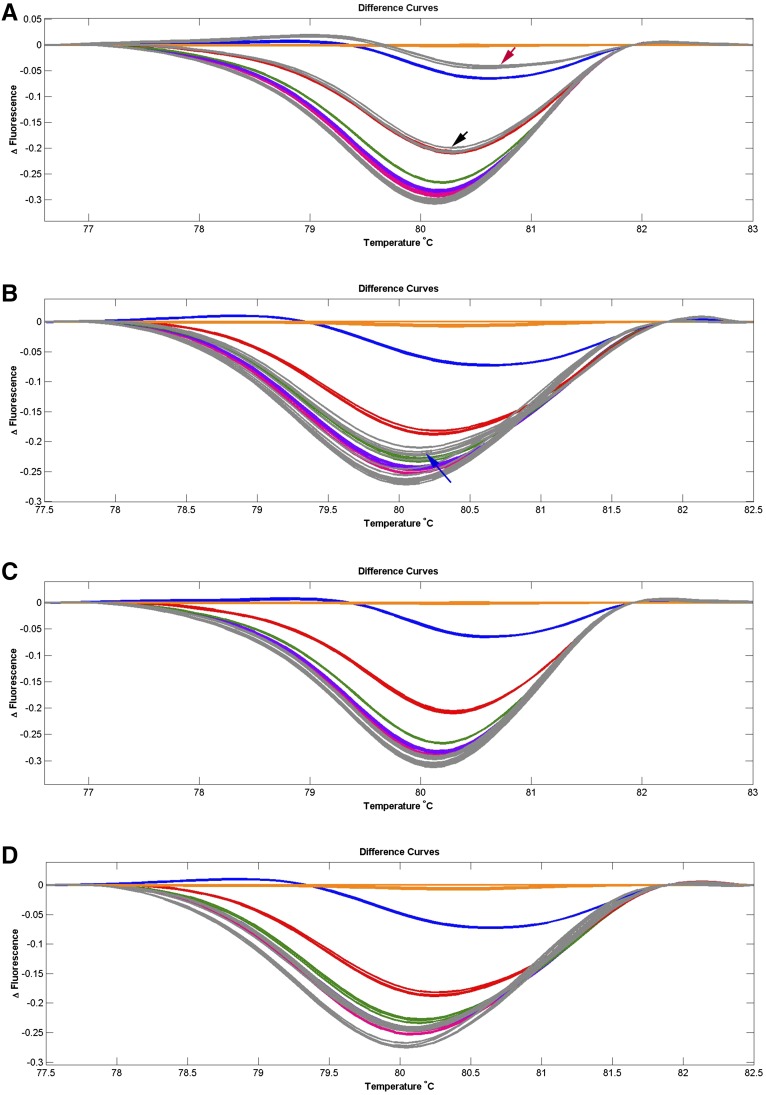
HRM analysis of *CYTB* PCR products from SCNT embryos. The different fluorescence curves were plotted by LightScanner HRM analysis software (Version 2.0). The dilutions of plasmids that carry PCR fragments at the ratio of donor cell:recipient oocyte of 50:50, 30:70, 10:90, 3:97, 1:99, and 0:100 are indicated by orange, blue, red, green, purple, and pink lines, respectively. SCNT embryos at the blastocyst stage are indicated in gray (*n* = 5/group). Each standard and sample were analyzed in triplicate. The different curves that overlap with or are below the pink line are considered to harbor only oocyte mtDNA. (A) Embryos derived from nondepleted cells. Two out of five embryos possessed donor cell mtDNA at levels of 10 and 30%, and are indicated by the black and red arrows, respectively. (B) Embryos derived from nondepleted cells in the presence of TSA. Two out of five embryos possessed donor cell mtDNA at levels of 3%, and are indicated by the blue arrow. (C) Embryos derived from depleted cells. All embryos harbored only oocyte mtDNA. (D) Embryos derived from depleted cells in the presence of TSA. All embryos harbored only oocyte mtDNA.

In order to determine the extent of the persistence of mtDNA from donor cells harboring mtDNA, we assessed embryos for mtDNA content. A total of two out of five SCNT blastocysts derived from embryos that were cultured in the absence of TSA possessed donor cell mtDNA at levels of 10 and 30% (Figure 3A). Donor cell mtDNA was also present in two out of five SCNT blastocysts derived from nondepleted cells cultured in the presence of TSA at levels of 3% (Figure 3B). However, when depleted cells were used as donor cells, blastocysts from embryos cultured in the presence and absence of TSA (Figure 3, C and D) harbored only oocyte mtDNA. As a result, SCNT blastocysts harboring recipient oocyte mtDNA only were successfully produced using mtDNA depleted donor cells in the presence and absence of TSA.

### Analysis of mtDNA copy number in SCNT embryos

**Figure 4:**
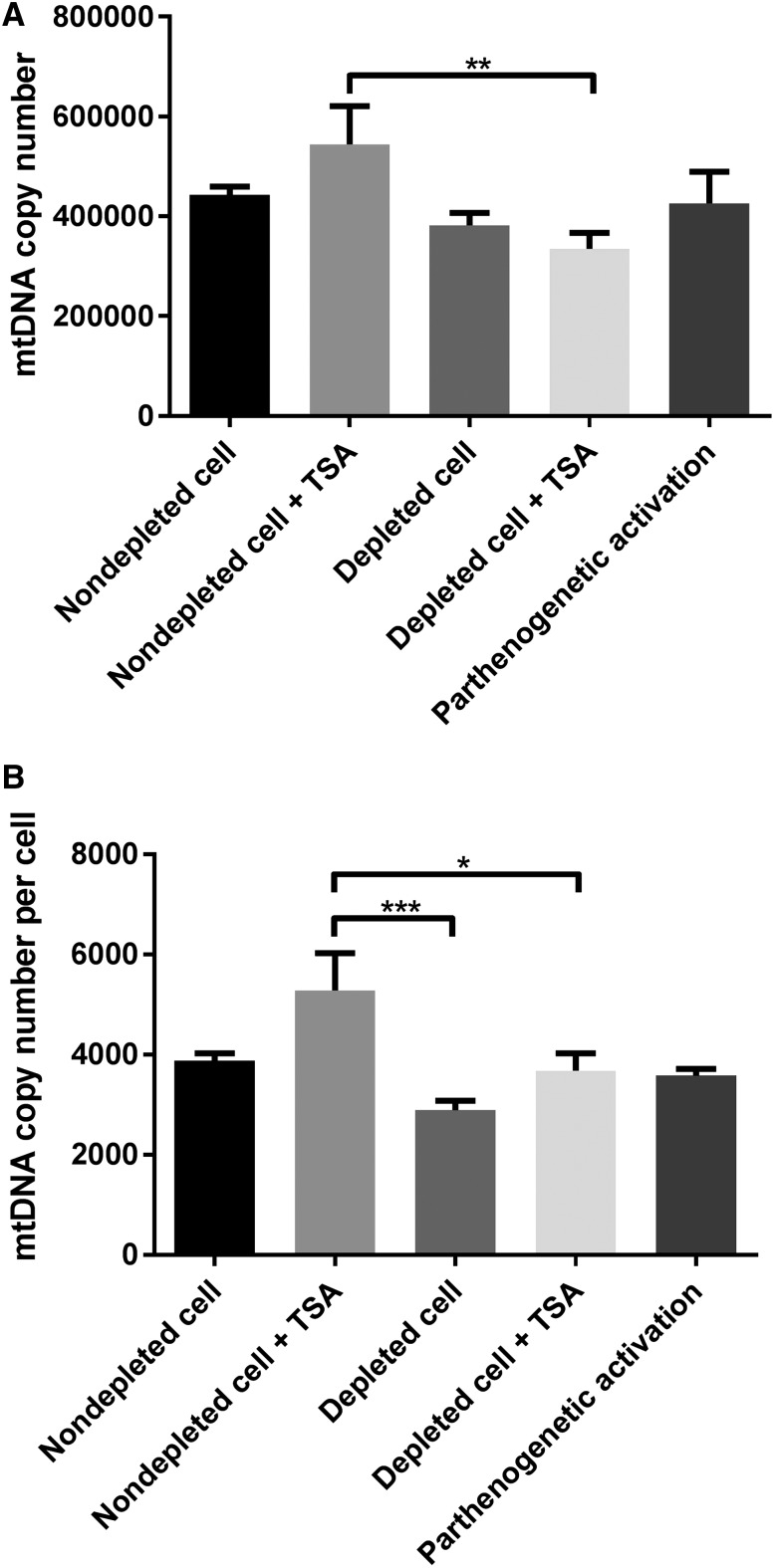
MtDNA copy number in SCNT embryos at the blastocyst stage. (A) mtDNA copy number per embryo. Mean values (±SEM) are shown. The differences were analyzed for significance by one-way ANOVA with Tukey’s multiple comparisons test using GraphPad Prism version 6.01. **P < 0.01. (B) mtDNA copy number per cell. Mean values (±SEM) are shown. The differences were analyzed for significance by one-way ANOVA with Tukey’s multiple comparisons test using GraphPad Prism version 6.01. *P < 0.05, ***P < 0.001, respectively.

As increased mtDNA has been associated with poor implantation rates and aneuploidy (Fragouli *et al.* 2015), we assessed mtDNA copy number in SCNT-derived blastocysts produced using *B. taurus* recipient oocytes. MtDNA copy number in blastocysts derived from depleted cells was not significantly different (382,359.75 ± 24,712.21, mean ± SEM) when compared with blastocysts from nondepleted cells (442,849.57 ± 16,509.73; Figure 4A). The presence of TSA had no significant effect on mtDNA copy number in SCNT blastocysts derived from depleted or nondepleted cells. However, mtDNA copy number for blastocysts derived from nondepleted cells cultured in the presence of TSA (544,364.12 ± 76,550.04) was significantly higher than that of blastocysts derived from depleted cells cultured in the presence of TSA (334,908.98 ± 31,807.74, *P* < 0.01). Furthermore, no significant difference was found in mtDNA copy number of SCNT embryos when compared with parthenogenetically activated (PA) embryos (426,203.31 ± 16,435.08). Moreover, when we assessed mtDNA copy number per cell at the blastocyst stage, depleting donor cells of mtDNA or their treatment with TSA had no significant effect on mtDNA copy number per cell (Figure 4B). However, mtDNA copy number per cell for embryos derived from nondepleted cells cultured in the presence of TSA was significantly higher than for blastocysts derived from depleted cells cultured in the presence and absence of TSA (Figure 4B).

Notably, mtDNA copy number for embryos at the blastocyst stage derived from depleted cells cultured in the presence (334,908.98 ± 31,807.74) or absence of TSA (382,359.75 ± 24,712.21) was significantly higher than that of oocytes at the MII stage (240,064.46 ± 8,153.74, *P* < 0.05 and *P* < 0.001, respectively, Figure S1A), as was the case for embryos derived from nondepleted cells (Figure S1B). These outcomes suggest that embryos derived from depleted cells have the potential to effectively regulate their mtDNA copy number during preimplantation development.

### Differentially expressed gene analysis in SCNT embryos

In order to determine whether restricting the mtDNA content of SCNT embryos to that of the recipient oocyte’s affects gene expression patterns at the blastocyst stage, we analyzed single embryos by RNA next generation sequencing. The number of genes expressed in each blastocyst was 8740 ± 56 (mean ± SEM) for blastocysts derived from nondepleted cells, 8816 ± 102 for blastocysts derived from nondepleted cells cultured in the presence of TSA, 8768 ± 99 for blastocysts derived from depleted cells, and 8821 ± 107 for blastocysts derived from depleted cells cultured in the presence of TSA. There was no significant difference in the number of genes expressed among each group.

**Figure 5:**
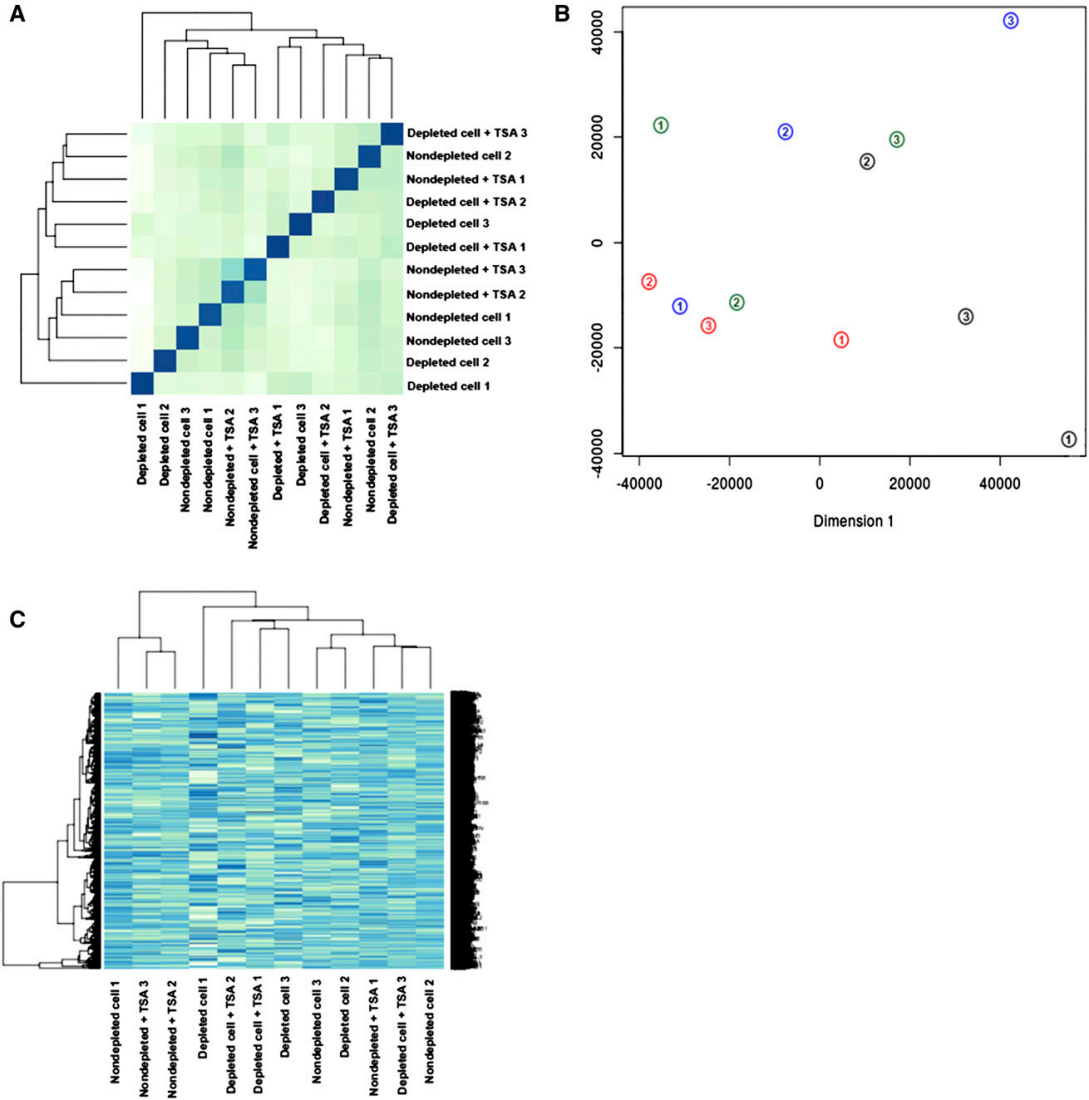
Global gene expression profiles of SCNT blastocysts. (A) A dendrogram generated from sample to sample distances. (B) Principle component analysis plot; individual blastocysts (*n* = 3/group) derived from nondepleted cells (green), nondepleted cells in the presence of TSA (blue), depleted cells (black), and depleted cells in the presence of TSA (red) are numbered. (C) Heat map of the 1000 most highly expressed genes, showing the correlations among each group of blastocysts. These blastocysts do not group according to their treatment.

**Figure 6:**
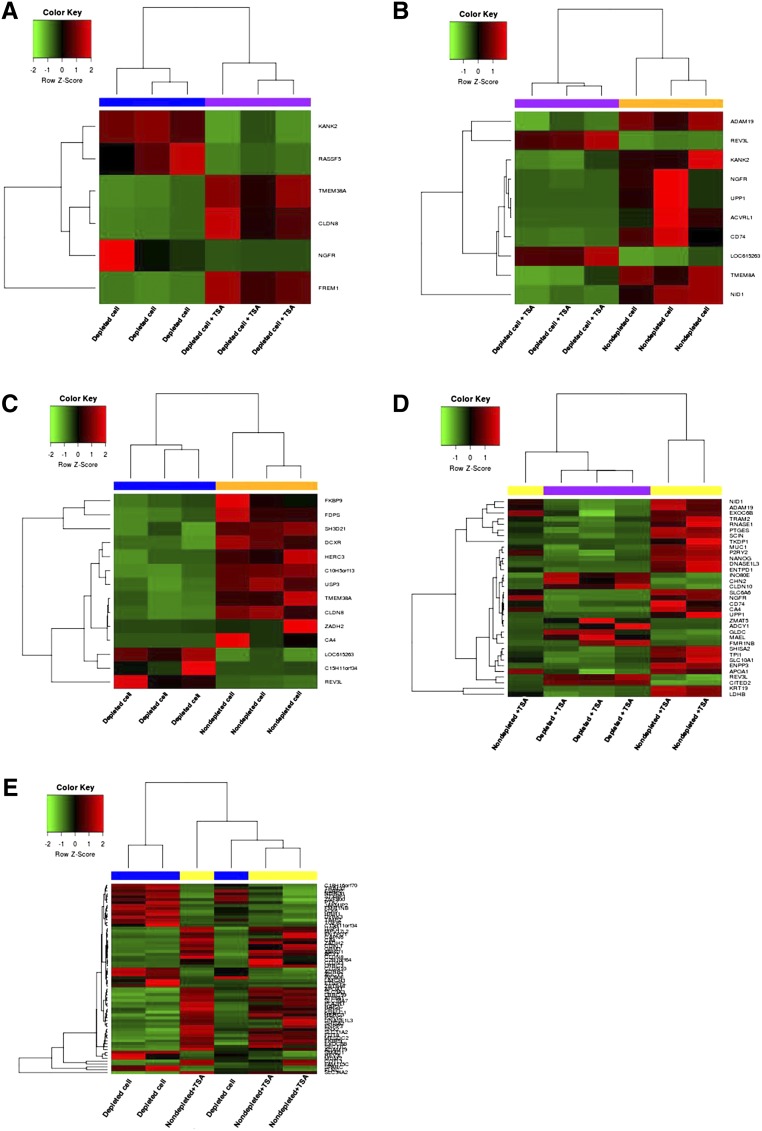
DEGs in SCNT embryos at the blastocyst stage. Heat map of differentially expressed genes between: (A) blastocysts derived from depleted cells in the presence and absence of TSA; (B) blastocysts derived from nondepleted cells in the absence of TSA and blastocysts derived from depleted cells in the presence of TSA; (C) blastocysts derived from nondepleted and depleted cells in the absence of TSA; (D) blastocysts derived from nondepleted and depleted cells in the presence of TSA; and (E) blastocysts derived from nondepleted cells in the presence of TSA, and blastocysts derived from depleted cells in the absence of TSA.

The dendrogram generated for sample to sample distances (Figure 5A), principle component analysis (Figure 5B), and the heat map of the 1000 most highly expressed genes (Figure 5C) showed that blastocysts were not necessarily grouped according to their treatment groups. However, the blastocysts derived from depleted cells and cultured in the presence of TSA clustered more closely together than the other groups. We used EdgeR to identify differentially expressed genes (DEGs) between each of the groups (cut-off level, FDR < 0.05). For the comparison between blastocysts derived from depleted cells cultured in the presence and absence of TSA, six genes were differentially expressed (FDR < 0.05). The presence of TSA resulted in decreased levels of gene expression for *NGFR*, *RASSF5*, and *KANK2*, while there were increased levels in expression for *TMEM38A*, *CLDN8*, and *FREM1* in embryos derived from just depleted cells (Figure 6A and Table S3 in File S4). For blastocysts derived from nondepleted cells cultured in the absence of TSA, compared with blastocysts derived from depleted cells cultured in the presence of TSA, eight out of 10 DEGs were upregulated. However, *REV3L* and *LOC615263* were downregulated for embryos derived from nondepleted cells (Figure 6B and Table S4 in File S4). Comparing blastocysts derived from depleted and nondepleted cells both cultured in the absence of TSA, *LOC615263*, *C15H11orf34*, and *REV3L* were upregulated for embryos from depleted cells while 11 other genes were downregulated (Figure 6C and Table S5 in File S4).

The comparison between blastocysts derived from depleted cells cultured in the presence of TSA and blastocysts from nondepleted cells cultured in the presence of TSA produced 35 DEGs (Table S6 in File S4). However, in this instance, the blastocysts generated from depleted cells clustered more closely together than the blastocysts from nondepleted cells (Figure 6D). Notably, *ADCY1*, *CHN2*, and *GLDC*, which are involved in DNA replication and repair and nucleic acid metabolism, were upregulated for embryos derived from depleted cells cultured in the presence of TSA (Figure 6D and Table S6). Indeed, the use of Ingenuity Pathway Analysis (IPA), where 34 out of 35 DEGs were annotated and clustered into four networks, highlighted the effects on DNA replication and repair and nucleic acid metabolism, as combined with small molecule biochemistry, this was the most affected network {IPA score, [−log(*P*-value)] = 43, Table S7 in File S4}. However, no upstream regulators were significantly activated (File S2).

Furthermore, there were 66 DEGs from the comparison between blastocysts from nondepleted cells cultured in the presence of TSA, and blastocysts derived from depleted cells cultured in the absence of TSA (Table S8 in File S4). The top 10 most significant DEGs are listed in Table 2. Indeed, 11 genes involved in cellular function and maintenance, molecular transport, and cell-to-cell signaling and interaction (*CA4*, *CLDN3*, *CLDN8*, *ENTPD1*, *FUT8*, *GRM1*, *LRRC59*, *P2RY2*, *RAP2C*, *SLC34A2*, and *SLC9A3*) were upregulated in blastocysts derived from nondepleted cells cultured in the presence of TSA (Figure 6E and Table S8). Again, the use of IPA annotated and clustered 63 out of the 66 DEGs into six networks, demonstrating that cellular function and maintenance, molecular transport, and cell-to-cell signaling and interaction was the most affected network (IPA score = 49, Table S9 in File S4). The predicted upstream regulators that were activated included *ENPP3*, *LIMCH1*, *NDRG1*, *SHISA2*, and *TIMP2*, which are involved in the pathway modulating ligand-dependent nuclear receptor, estrogen receptor 2 (ESR2, File S3). Interestingly, no significant DEGs were identified for blastocysts derived from nondepleted cells cultured in the presence and absence of TSA.

**Table 2:** List of the top 10 most significant DEGs from the comparison between SCNT blastocysts derived from nondepleted cells cultured in the presence of TSA, and depleted cells cultured in the absence of TSA (*n* = 66). Fold changes are relative to SCNT blastocysts derived from depleted cells in the absence of TSA

No.	Gene Symbol	Gene ID	Gene Name	Chr	Fold Change	Regulation	FDR	Type
1	*C2H1orf64*	NM_001205359.1	Chromosome 2 open reading frame, human C1orf64	2	26.52983	Up	0.001904	NA
2	*EXOC6B*	NM_001076416.1	Exocyst complex component 6B	11	2.325931	Up	0.001904	Other
3	*ADCY1*	NM_174229.2	Adenylate cyclase type 1	4	679.3225	Down	0.001904	Peptidase
4	*TGFBI*	NM_001205402.1	Transforming growth factor beta induced	7	7.94732	Down	0.001904	Other
5	*CA4*	NM_173897.1	Carbonic anhydrase IV	19	418.4826	Up	0.002735	Enzyme
6	*KLK6*	NM_001101140.2	Kallikrein related peptidase 6	18	4.195303	Down	0.003356	Peptidase
7	*REV3L*	NM_001206172.1	REV3-like, polymerase (DNA directed), zeta, catalytic subunit	9	2.357427	Down	0.003356	Enzyme
8	*FMR1NB*	NM_001102262.2	Fragile X mental retardation 1 neighbor	X	18.72273	Down	0.003369	Other
9	*DNASE1L3*	NM_001205724.1	Deoxyribonuclease I-like 3	22	91.94144	Up	0.003886	Enzyme
10	*RAP2C*	NM_001075700.1	RAP2C, member of RAS oncogene family	X	2.888357	Up	0.003899	Enzyme

**Figure 7:**
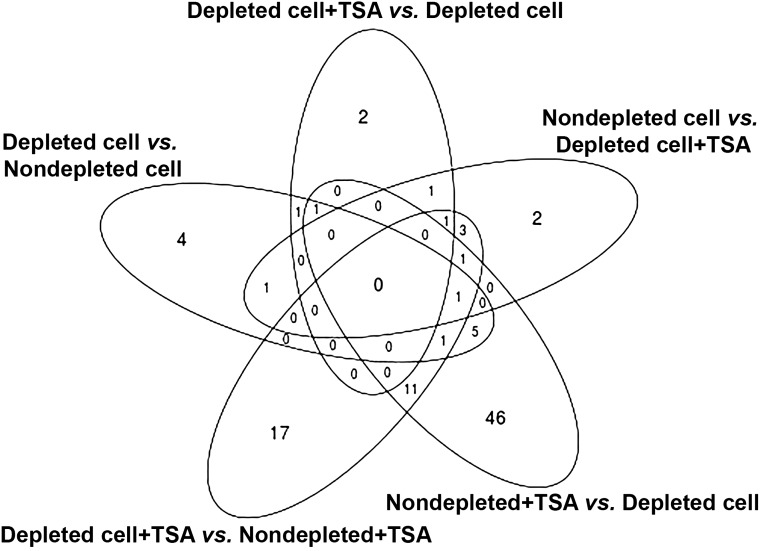
Venn diagram showing the commonly differential expressed genes among each comparison group. Five comparisons are shown. There are no commonly expressed genes for all of the comparison groups.

The Venn diagram in Figure 7 shows the number of overlapping DEGs in SCNT embryos, which are documented in Table 3. There were no commonly DEGs among the five comparison groups. Interestingly, depletion of donor cell mtDNA affected the expression levels of *REV3L*, *ADAM19*, *CA4*, *FKBP9*, *HERC3*, *USP3*, *ZADH2*, *ADCY1*, *APOA1*, *CLDN10*, *DNASE1L3*, *ENPP3*, *ENTPD1*, *EXOC6B*, *INO80E*, *SHISA2*, and *P2RY2*, which are associated with the cell cycle, cell-to-cell signaling and interaction, and cellular growth and proliferation network. The combination of depleted donor cells, and the presence of TSA affected expression of genes (*NGFR*, *KANK2*, *CD74*, *NID1*, and *UPP1*) involved in the cell-to-cell signaling and interaction, hematological system development and function, and immune cell trafficking networks. Moreover, the presence of TSA in embryos derived from depleted cells positively modulated the expression patterns of *CLDN8* and *TMEM38A* (Table 4).

**Table 3:** Common DEGs among the five comparisons

Comparison	Gene Symbol	Gene ID	Gene Name	Chr
Nondepleted cell *vs.* depleted cell+TSA Depleted cell *vs.* nondepleted cell Depleted cell+TSA *vs.* nondepleted cell+TSA Nondepleted cell+TSA *vs.* depleted cell	*REV3L*	NM_001206172.1	REV3-like, polymerase (DNA directed), zeta, catalytic subunit	9
Depleted cell+TSA *vs.* depleted cell Nondepleted cell *vs.* depleted cell+TSA Depleted cell+TSA *vs.* non-depleted cell+TSA	*NGFR*	NM_001102478.2	Nerve growth factor receptor	19
Nondepleted cell *vs.* depleted cell+TSA Depleted cell+TSA *vs.* nondepleted cell+TSA Nondepleted cell+TSA *vs.* depleted cell	*ADAM19*	NM_001075475.2	ADAM metallopeptidase domain 19	7
Depleted cell *vs.* nondepleted cell Depleted cell+TSA *vs.* nondepleted cell+TSA Nondepleted cell+TSA *vs.* depleted cell	*CA4*	NM_173897.1	Carbonic anhydrase IV	19
Depleted cell+TSA *vs.* depleted cell Depleted cell *vs.* nondepleted cell Nondepleted cell+TSA *vs.* depleted cell	*CLDN8*	NM_001098096.1	Claudin 8	1
				
Nondepleted cell *vs.* depleted cell+TSA Depleted cell *vs.* nondepleted cell	*LOC615263*	NM_001103298.1	Uncharacterized LOC615263	2
Depleted cell+TSA *vs.* depleted cell Depleted cell *vs.* nondepleted cell	*TMEM38A*	NM_001083463.1	Transmembrane protein 38A	7
Depleted cell+TSA *vs.* depleted cell Nondepleted cell *vs.* depleted cell+TSA	*KANK2*	NM_001076531.1	KN motif and ankyrin repeat domains 2	7
Nondepleted cell *vs.* depleted cell+TSA Depleted cell+TSA *vs.* nondepleted cell+TSA	*CD74*	NM_001034735.1	CD74 molecule, major histocompatibility complex, class II invariant chain	7
	*NID1*	NM_001101155.1	Nidogen 1	28
	*UPP1*	NM_001098976.2	Uridine phosphorylase 1	4
Depleted cell *vs.* nondepleted cellNondepleted cell+TSA *vs.* depleted cell	*C15H11orf34*	NM_001113538.1	Chromosome 15 open reading frame, human C11orf34	15
	*FKBP9*	NM_001046372.2	FK506 binding protein 9	4
	*HERC3*	NM_001083663.1	HECT and RLD domain containing E3 ubiquitin protein ligase 3	6
	*USP3*	NM_001102123.1	Ubiquitin specific peptidase 3	10
	*ZADH2*	NM_001075964.1	Zinc binding alcohol dehydrogenase domain containing 2	24
Depleted cell+TSA *vs.* nondepleted cell+TSA Nondepleted cell+TSA *vs.* depleted cell	*ADCY1*	NM_174229.2	Adenylate cyclase type 1	4
	*APOA1*	NM_174242.3	Apolipoprotein A-I	15
	*CLDN10*	NM_001014857.1	Claudin 10	12
	*DNASE1L3*	NM_001205724.1	Deoxyribonuclease I-like 3	22
	*ENPP3*	NM_001075923.2	Ectonucleotide pyrophosphatase/phosphodiesterase 3	9
	*ENTPD1*	NM_174536.2	Ectonucleoside triphosphate diphosphohydrolase 1	26
	*EXOC6B*	NM_001076416.1	Exocyst complex component 6B	11
	*FMR1NB*	NM_001102262.2	Fragile X mental retardation 1 neighbor	X
	*INO80E*	NM_001046544.1	INO80 complex subunit E	25

**Table 4:** List of genes affected by donor cell mtDNA depletion and the presence of TSA

Treatment	Gene List
Depleted cells	*REV3L*, *ADAM19*, *CA4*, *LOC615263*, *C15H11orf34*, *FKBP9*, *HERC3*, *USP3*, *ZADH2*, *ADCY1*, *APOA1*, *CLDN10*, *DNASE1L3*, *ENPP3*, *ENTPD1*, *EXOC6B*, *FMR1NB*, *INO80E*, *P2RY2*, *SHISA2*
Depleted cells and TSA treatment	*NGFR*, *KANK2*, *CD74*, *NID1*, *UPP1*
TSA treatment	*CLDN8*, *TMEM38A*

**Figure 8:**
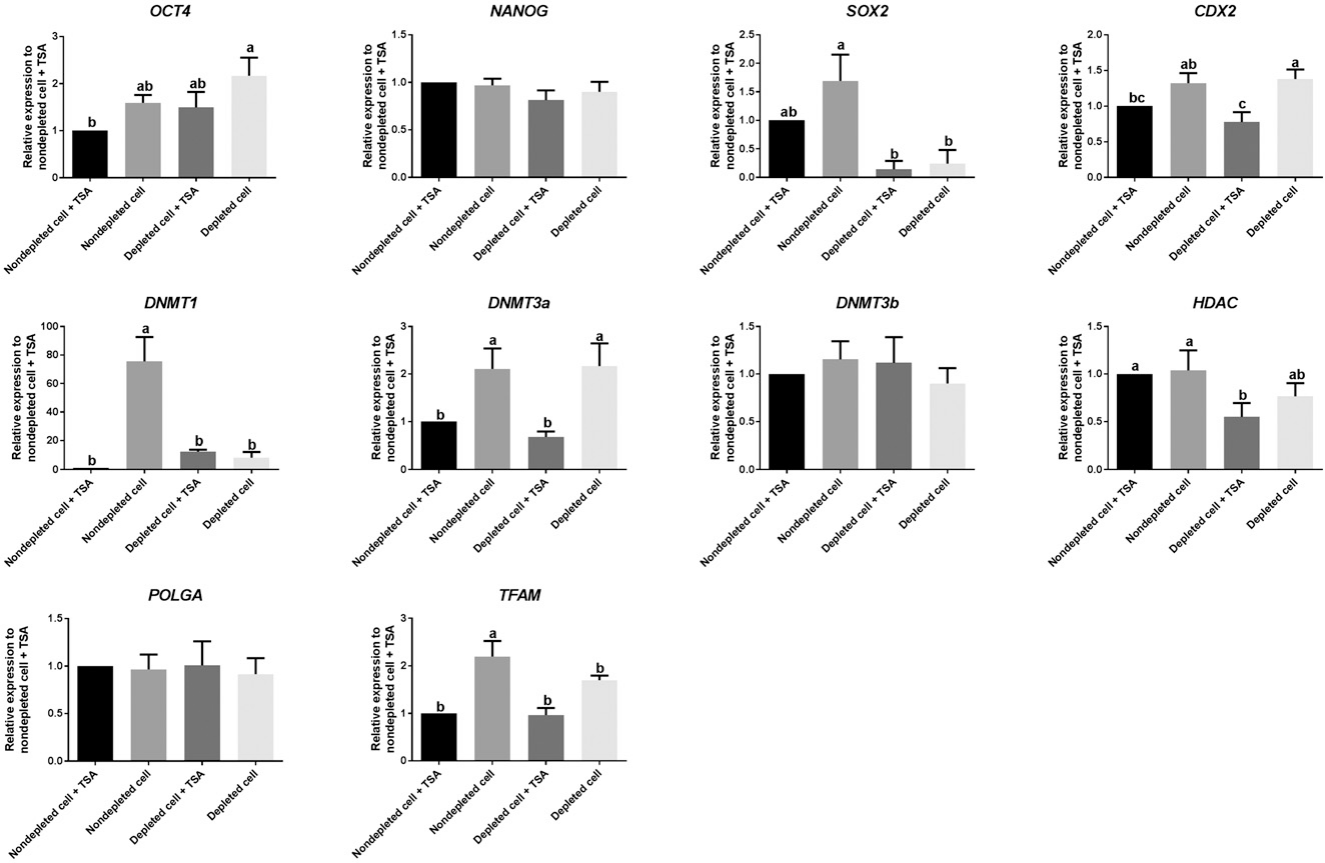
Real time PCR to determine levels of expression of genes involved in pluripotency and differentiation, epigenetic regulation, and mtDNA replication in SCNT-derived blastocysts. Values represent mean ± SEM; *n* = 3/group. Different superscripts within a column indicate significant differences (*P* < 0.05, ANOVA with Duncan’s multiple range test).

As the compartmentalization of the inner cell mass and trophectoderm is essential to blastocyst formation, we assessed each group for the expression of the transcription factors associated with maintaining the inner cell mass (*OCT4*, *SOX2*, and *NANOG*) and establishing the trophectoderm (*CDX2*) by real time PCR. There was subtle variability among each set of blastocysts, with fold changes <2. However, expression levels for blastocysts generated from depleted cells in the presence of TSA were closer to blastocysts derived from nondepleted cells in the presence of TSA than for blastocysts generated from depleted and nondepleted cells in the absence of TSA. There were similar outcomes for the modulators of DNA demethylation, namely *DNMT1* and *DNMT3a*. However, there were no differences for *DNMT3b* and a marginal difference for *HDAC*. In terms of the key mtDNA replication factors, again, blastocysts from nondepleted cells cultured in the absence of TSA exhibited significantly higher levels of expression of mitochondrial transcription factor A (*TFAM*). The late expression (cycle number 43–45) of DNA Polymerase Gamma A (*POLGA*) reflects controlled levels of mtDNA replication (Figure 8). Overall, blastocysts generated from depleted cells cultured in the presence of TSA exhibited a more consistent pattern of gene expression for key regulators of early development.

## Discussion

To the best of our knowledge, this is the first report of the use of ddC to deplete donor cells of their mtDNA prior to SCNT. Ethidium bromide has been used to deplete mtDNA from donor cells to generate both sheep and goat-sheep embryos (Lloyd *et al.* 2006) and sheep offspring (Lee *et al.* 2010). However, ethidium bromide is highly toxic as a mutagen, and also a possible carcinogen or teratogenic (Waring 1965). The nucleoside reverse transcriptase inhibitor, ddC, has been used widely to suppress HIV replication, and affects the interaction of POLG with the mitochondrial genome (Brinkman and Kakuda 2000). The actions of ddC are more specific as an inhibitor of mtDNA replication than its counterpart ethidium bromide (Brown and Clayton 2002), as it binds specifically to POLG, which effects only replication of mtDNA, and not the nuclear DNA polymerases, namely DNA polymerase alpha, delta, and epsilon (reviewed by Brinkman *et al.* (1998)). Therefore, ddC was chosen to deplete mtDNA from donor cells.

However, as a note of caution, previous reports have shown that ddC can decrease cellular lipids, increase apoptosis, and induce mitochondrial depolarization (Walker *et al.* 2006). Moreover, as loss of function in the mitochondrial electron transport chain in mtDNA depleted cells can inhibit the *de novo* synthesis of pyrimidines, resulting in these cells failing to proliferate (King and Attardi 1989), it is important to add substrates such as uridine to the culture medium to ensure sufficient pyrimidines are produced (Grégoire *et al.* 1984). Indeed, uridine is used frequently in mtDNA depletion experiments in respect of both ethidium bromide (Lloyd *et al.* 2006; Lee *et al.* 2010) and ddC (Keilbaugh *et al.* 1993; Nelson *et al.* 1997). Nevertheless, mtDNA-depleted cells continue to have lower proliferation rates in the presence of uridine (Lloyd *et al.* 2006; Bowles *et al.* 2007), which is advantageous, as reduced cell proliferation in donor cells is normally mediated through serum starvation prior to SCNT in order that they are synchronized to the G0/G1 phase (Campbell *et al.* 1996; Wilmut *et al.* 1997). Furthermore, from our CGH microarray results, it is evident that depleting donor cells of their mtDNA, by culturing in ddC with uridine for 30 d, does not alter their chromosomal stability.

The use of mtDNA depleted cells as donor cells for SCNT resulted in all embryos harboring only oocyte mtDNA, as is the case following sperm-mediated fertilization (Sato and Sato 2012). However, the mixing of two populations of mtDNA, namely from the donor cell and the recipient oocyte, was identified in embryos derived from nondepleted cells at levels ranging from 0 to 30% for donor cell mtDNA. Notably, replication of donor cell mtDNA represented a 0- to 886-fold increase from the amount introduced into the recipient oocyte for SCNT blastocysts derived from nondepleted cells. This demonstrates the random nature of the transmission of donor cell mtDNA if cells are not depleted. As a previous report has shown, SCNT embryos produced by donor cells harboring a full complement of mtDNA, or were partially or almost completely depleted of mtDNA also possessed two populations of mtDNA (Lloyd *et al.* 2006), demonstrating that even low levels of mtDNA can be transmitted. These somatic cell mitochondria are likely to be detrimental to development as demonstrated by the introduction of somatic cell mitochondrial isolates into mouse oocytes (Takeda *et al.* 2005, 2012). Furthermore, there is a considerable beneficial effect from using depleted cells as donor cells since cell number in SCNT blastocysts was increased when using partially depleted cells (Lloyd *et al.* 2006; Lee *et al.* 2010), as is the case for our cattle embryos. In addition, mtDNA depletion results in an increase in the rates of pregnant females and live born offspring in sheep (Lee *et al.* 2010). These outcomes demonstrate the importance of depleting donor cells to promote developmental outcomes, and to ensure that the resultant offspring inherit their mtDNA in a true maternal manner.

TSA increased developmental rates to the blastocyst stage for embryos derived from depleted cells to levels equivalent to that of nondepleted cells. However, no beneficial effects were observed for the embryos derived from nondepleted cells. Several reports show that the effect of TSA on embryo development is controversial in different species (Monteiro *et al.* 2010), and tends to be dependent on donor cell type (Kishigami *et al.* 2006; Martinez-Diaz *et al.* 2010). However, to date, there has been no report of the use of TSA on embryos derived from mtDNA-depleted cells. A previous report in sheep showed that two live offspring were successfully produced by using mtDNA-depleted cells as the donor cells for SCNT (Lee *et al.* 2010). Although Lee *et al.* (2010) used a different depletion reagent (ethidium bromide) to that used in this study, they showed that embryos derived from depleted cells can develop to term and result in viable lambs. The effects of TSA on pre- and postimplantation development of embryos are still debatable. Although previous reports have shown that TSA had detrimental effects on embryo development (Tsuji *et al.* 2009) and the health of the offspring (Meng *et al.* 2009; Zhao *et al.* 2010), healthy live offspring derived from SCNT embryos cultured in the presence of TSA have been produced successfully in many species such as mice (Kishigami *et al.* 2006, 2007), cattle (Sawai *et al.* 2012; Srirattana *et al.* 2012), and pigs (Li *et al.* 2008). Taken together, there is the potential to generate live offspring from the combination of mtDNA-depleted cells and TSA supplementation.

Normally, mtDNA replication does not take place in embryos until the blastocyst stage (May-Panloup *et al.* 2005; Spikings *et al.* 2007). Therefore, transferring mtDNA depleted cells does not have negative effects in term of mtDNA copy number in oocytes. Moreover, these embryos have the potential to effectively regulate their mtDNA copy number, as the mtDNA copy number in blastocysts derived from depleted cells was ∼1.5 times higher when compared with that of MII oocytes. Depleting mtDNA from donor cells also has a beneficial effect on controlling the level of mtDNA copy number in blastocysts, as the blastocysts derived from depleted cells maintained their mtDNA copy number at a level that is within range for postimplantation development. However, mtDNA copy number per cell in blastocysts derived from nondepleted cells in the presence of TSA was significantly higher than that for depleted cells cultured in the presence and absence of TSA. Notably, human fertilized blastocysts that have a higher mtDNA copy number than the relative cut-off threshold are more prone to aneuploidy (Fragouli *et al.* 2015), and lower viability and implantation rates (Diez-Juan *et al.* 2015).

Our RNA-sequencing data produced a small number of genes that were differentially expressed among each of the comparisons (cut-off level, FDR < 0.05). However, this finding revealed that depleting mtDNA from donor cells prior to SCNT and culture in the presence of TSA after SCNT changed the expression profiles of the resultant embryos. Depleting donor cell mtDNA prior to SCNT affects expression patterns of genes associated with cell death and survival in SCNT embryos at the blastocyst stage. Interestingly, *REV3L*, which is involved in the DNA repair process, was upregulated in embryos derived from depleted cells. Loss of *REV3L* causes chromosomal instability, and failure of embryogenesis in mouse (Lange *et al.* 2016). The presence of TSA positively modulated the expression levels of *CLDN8*, *TMEM38A*, and *FREM1* in embryos derived from depleted cells. *CLDN8* is one of the Claudin family of tight junction membrane proteins, which play important roles in maintaining blastocoel shape though hydrostatic pressure. Removal of Claudins from the trophectoderm of mouse embryos causes collapsing of the blastocyst and disruption of embryonic development (Moriwaki *et al.* 2007). *TMEM38A*, also known as trimeric intracellular cation channel (*TRIC*) type A, is a monovalent cation channel that is required for maintenance of rapid intracellular calcium release. Double knockout mice lacking *TRIC* subtype A and B exhibited cardiac problems, leading to embryonic lethality at embryonic day 10.5 (Yazawa *et al.* 2007). Moreover, *FREM1* is generally expressed in the developing embryo, and is essential for the normal adhesion of the embryonic epidermis (Smyth *et al.* 2004). Therefore, TSA has beneficial effects on embryos derived from depleted cells in terms of increasing expression levels of genes involved in embryo development. Moreover, blastocysts derived from depleted cells cultured in the presence of TSA exhibited different gene expression profiles, and are clustered more closely together than blastocysts derived from nondepleted cells in the presence of TSA. However, blastocysts derived from nondepleted cells in the presence of TSA had a higher mtDNA copy number and possessed donor cell mtDNA. These two differences are likely to be the mediators of the changes in gene expression as they are the only two anomalies between the two cohorts.

The 66 DEGs from the comparison between embryos derived from nondepleted cells in the presence of TSA, and from depleted cells in the absence of TSA, showed that these two sets of blastocysts were very different. These results correlated with the significant differences in mtDNA copy number per cell in blastocysts and levels of expression of *OCT4* and *CDX2* from real-time RT-PCR. Notably, two DEGs, *FAM115C* and *SLC16A7*, which have been reported to be key genes expressed in cattle *in vivo*-derived embryos at the blastocyst stage (Jiang *et al.* 2014), were upregulated in embryos derived from nondepleted cells in the presence of TSA. Upstream regulator analysis showed that ESR2, which plays an important role in embryo development (Bondesson *et al.* 2015), was activated in embryos derived from nondepleted cells in the presence of TSA.

Moreover, our RNA-sequencing data provided new insights with the unannotated genes such as *LOC615263* and *C15H11orf34* being upregulated in embryos derived from depleted cells. A previous report showed that expression of *LOC615263* was upregulated in cattle embryos at day 13 after the transfer of *in vitro*-derived blastocysts when compared with *in vivo*-derived blastocysts (Clemente *et al.* 2011). Other unannotated genes were also identified in this study such as *C10H5orf13*, *C18H16orf70*, and *C2H1orf64*.

From real time RT-PCR, small fold changes (<2) between each of the cohorts of embryos showed that the expression patterns of embryos derived from depleted and nondepleted cells were slightly different. The expression levels of *SOX2*, *DNMT1*, and *TFAM* were significantly lower in embryos derived from depleted cells when compared with nondepleted cells. When focusing on blastocysts derived from depleted cells, TSA had no effect on *OCT4*, *SOX2*, and *NANOG*, which are key regulators of the inner cell mass. In support of this, the use of TSA on donor cells prior to SCNT was shown to have no effect on the expression of *OCT4* and *NANOG* in cattle SCNT blastocysts (Hosseini *et al.* 2016). Moreover, the levels of expression of *CDX2* and *DNMT3a*, as well as cell number in embryos derived from depleted cells, were decreased after culture in TSA. Consequently, it appears that TSA appropriately modulates the expression profiles of transcription factors involved in establishing blastocyst formation, especially as the expression profiles of blastocysts derived from depleted cells cultured in the presence of TSA were closer to blastocysts derived from nondepleted cells cultured in the presence of TSA than for any other group.

The results from this study showed that embryos derived from depleted cells and cultured in the presence of TSA seem to be normal when compared with that of nondepleted cells. As only one cell line from an individual cow was used to undertake all the work in the current investigations, the effects of mtDNA depletion in SCNT embryos might be cell line specific. However, we were able to determine that two other cell lines were also competent for generating blastocyst stage embryos. Nevertheless, the effects of mtDNA depletion on other cell lines would need to be compared in terms of the outcomes related to gene expression changes.

In conclusion, depleting mtDNA from the donor cells prior to SCNT has beneficial effects in terms of SCNT embryos inheriting only one population of mtDNA, namely from their oocytes. This increases cell number and controls mtDNA copy number at the blastocyst stage. Analyses of DEGs showed that the expression patterns of blastocysts derived from depleted cells were different to that of nondepleted cells. Based on network analysis, the DEGs between these two groups of embryos were primarily involved in embryonic development. Moreover, the presence of TSA significantly enhanced blastocyst formation rates for embryos derived from depleted cells, which positively modulated expression patterns of genes associated with the inner cell mass and DNA methylation, as well as embryonic development.

## Supplementary Material

Supplemental material is available online at www.g3journal.org/lookup/suppl/doi:10.1534/g3.117.042655/-/DC1.
