# Supplementary figures and images for: Manipulating the Mitochondrial Genome To Enhance Cattle Embryo Development
Source: G3 (Bethesda). 2017 May 8;7(7):2065–80. doi: 10.1534/g3.117.042655 (PMC5499117; doi:10.1534/g3.117.042655)

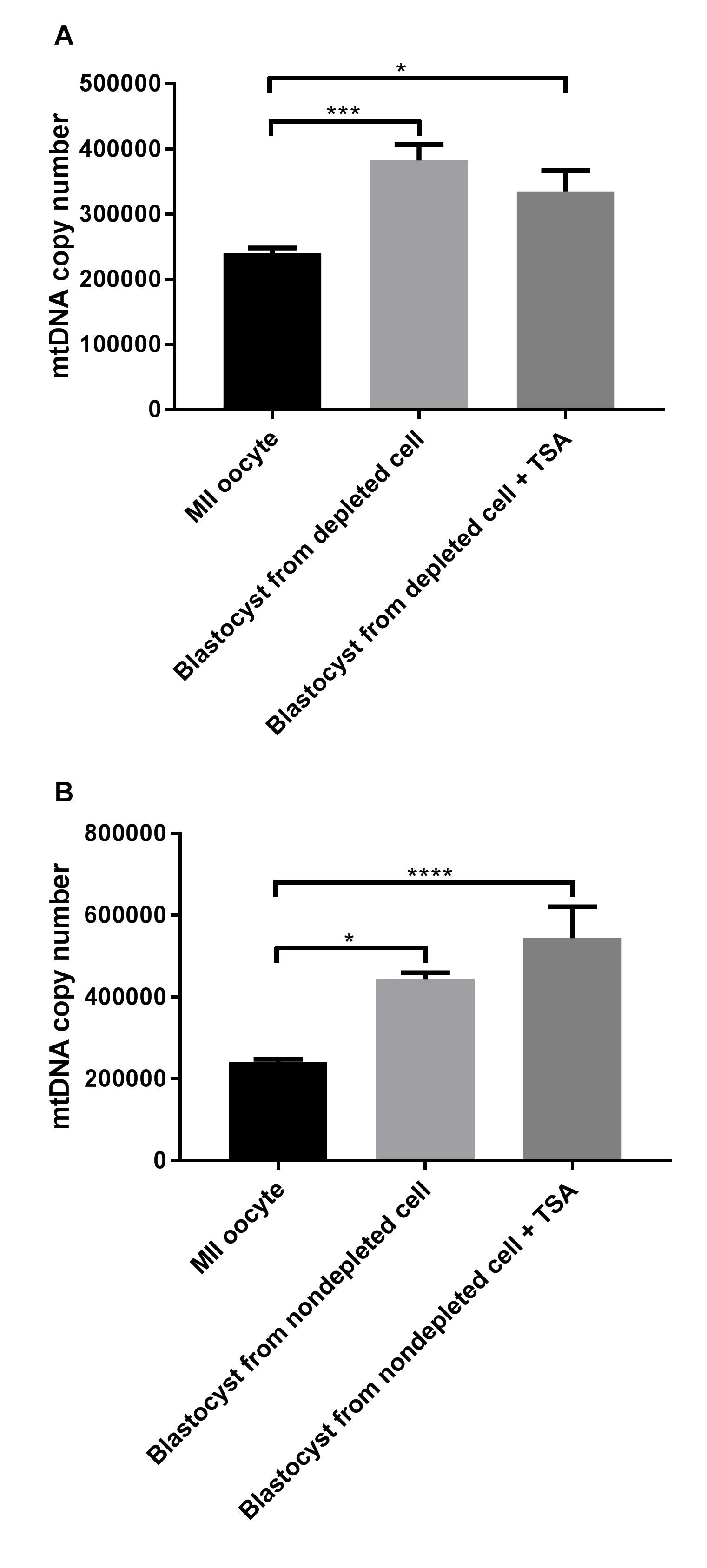

Supplement: Supplementary file 1 [file 2065FigureS1.jpg]
