# Supplementary material for: Manipulating the Mitochondrial Genome To Enhance Cattle Embryo Development
Source: G3 (Bethesda). 2017 May 8;7(7):2065–80. doi: 10.1534/g3.117.042655 (PMC5499117; doi:10.1534/g3.117.042655)
Supplement: Supplementary file 5 [file 2065FileS4.docx]

**Table S1.** Primers used for amplification of mtDNA and for markers of blastocyst gene expression.

| **Purpose** | **Primer region** | **Sequences 5’-3’** |
| --- | --- | --- |
| Real time PCR | *CYTB* | F: CCTCTGTTACCCATATCTGCCG |
|  |  | R: GTGCCGATGTATGGGATTGC |
|  | *ND2* | F: TATACGACTCACGTATTCTACC |
|  |  | R: CTTTGAAGGCTCTTGGTCTG |
|  | *POU* | F: CCCAGTGGAAGAGGGGGTGA |
|  |  | R: ACCCCACACCGGCTACTCTT |
|  | *ACTB* | F: GGATGAGGCTCAGAGCAAGAGA |
|  |  | R: TCGTCCCAGTTGGTGACGAT |
|  | *OCT4* | F: CCACCCTGCAGCAAATTAGC |
|  |  | R: CCACACTCGGACCACGTCTT |
|  | *NANOG* | F: CACCCATGCCTGAAGAAAGT |
|  |  | R: GGCTTGTGGAAGAATCAGGA |
|  | *SOX2* | F: CACAACTCGGAGATCAGCAA |
|  |  | R: CGGGGCCGGTATTTATAATC |
|  | *CDX2* | F: GCAAAGGAAAGGAAAATCAACAA |
|  |  | R: GGGCTCTGGGACGCTTCT |
|  | *DNMT1* | F: TTCTGCAGCAAGAAGAGCAA |
|  |  | R: TCGCTCCAGTAAAGCAGGTT |
|  | *DMNT3a* | F: CATCAAAGAAGACCCCTGGA |
|  |  | R: GAGGATCGAATTCCTGGTCA |
|  | *DNMT3b* | F: CGGCCTATTCGAGTCTTGTC |
|  |  | R: AACGGCAATGGACTCTTCAC |
|  | *HDAC* | F: ATCGGTTAGGTTGCTTCAATCTG |
|  |  | R: GTTGTATGGAAGCTCATTAGGGA |
|  | *POLGA* | F: AGTCCCAGAGCAAAGCCAAC |
|  |  | R: TGTTGCCCTTGACAAACAGC |
|  | *TFAM* | F: CAAATGATGGAAGTTGGACG |
|  |  | R: AGCTTCCGGTATTGAGACC |
| HRM | *CYTB* | F: GTAATCCTTCTGCTCACAGTAA |
|  |  | R: TCCTCATGGTAGGACGTATC |
| Long-range PCR | F1 | F1: GCAGTAGCACAAACAATCTCATAC |
|  | R1 | R1: AGGATGAGCATAGTAATGAGGAATAG |
|  | F1 | F2: GTAATCACCGCCCTATATTCTCTATAC |
|  | R2 | R2: GGGATGATGGCGAGTATATTTATTTC |
| Conventional PCR | D-loop | F: CACCATCAACCCCCAAAGCT |
|  |  | R: CCTGAAGAAAGAACCAGATG |

**Table S2.** Developmental potential of cattle embryos derived from mtDNA depleted Holstein donor cells from 2 different individuals in the presence of Trichostatin A.

| **Donor cell** | **Fused (%)** | **No. embryos** | **Cleaved  (%)*** | **No. (%)* embryo developed to** | | |
| --- | --- | --- | --- | --- | --- | --- |
|  |  |  |  | **8-Cell** | **Morulae** | **Blastocysts** |
| Holstein No. 2 | 45/48  (93.8 ± 1.3) | 40 | 35  (87.5 ± 3.8) | 25  (62.5 ± 6.3) | 11  (27.5 ± 0.3) | 5  (12.5 ± 1.3) |
| Holstein No. 3 | 44/49  (89.8 ± 5.1) | 39 | 33  (84.6 ± 6.3) | 16  (41.0 ± 2.0) | 8  (20.5 ± 1.6) | 6  (15.4 ± 1.2) |

*Percentages calculated from the number of embryos that cleaved and developed to each stage.

Values are mean ± SEM.

Two replicates.

**Table S3.** Differentially expressed genes from the comparison of SCNT blastocysts derived from depleted cells cultured in the presence and absence of TSA (n = 6). Fold changes are relative to SCNT blastocysts derived from depleted cells cultured in the absence of TSA.

| **Gene Symbol** | **Gene ID** | **Gene name** | **Chr** | **Fold change** | **Regulation** | **FDR** | **Type** |
| --- | --- | --- | --- | --- | --- | --- | --- |
| *FREM1* | NM_001192995.1 | FRAS1 related extracellular matrix 1 | 8 | 15.63554 | Up | 0.00285013 | other |
| *CLDN8* | NM_001098096.1 | Claudin 8 | 1 | 14.86327 | Up | 0.004227155 | other |
| *TMEM38A* | NM_001083463.1 | Transmembrane protein 38A | 7 | 8.306495 | Up | 0.014774177 | ion channel |
| *NGFR* | NM_001102478.2 | Nerve growth factor receptor | 19 | 24.10004 | Down | 0.014774177 | transmembrane receptor |
| *RASSF5* | NM_001113760.2 | Ras association (RalGDS/AF-6) domain family member 5 | 16 | 7.589196 | Down | 0.006665638 | other |
| *KANK2* | NM_001076531.1 | KN motif and ankyrin repeat domains 2 | 7 | 4.769194 | Down | 0.014774177 | transcription regulator |

**Table S4.** Differentially expressed genes from the comparison of SCNT blastocysts derived from nondepleted cells cultured in the absence of TSA and depleted cells cultured in the presence of TSA (n = 10). Fold changes are relative to SCNT blastocysts derived from depleted cells cultured in the presence of TSA.

| **Gene Symbol** | **Gene ID** | **Gene name** | **Chr** | **Fold change** | **Regulation** | **FDR** | **Type** |
| --- | --- | --- | --- | --- | --- | --- | --- |
| *UPP1* | NM_001098976.2 | Uridine phosphorylase 1 | 4 | 265.9733 | Up | 0.0475261 | enzyme |
| *ACVRL1* | NM_001083479.1 | Activin A receptor type IL | 5 | 184.9281 | Up | 0.0475261 | transmembrane receptor |
| *CD74* | NM_001034735.1 | CD74 molecule, major histocompatibility complex, class II invariant chain | 7 | 28.98734 | Up | 3.112E-06 | transmembrane receptor |
| *NGFR* | NM_001102478.2 | Nerve growth factor receptor | 19 | 28.33119 | Up | 0.0077679 | transmembrane receptor |
| *NID1* | NM_001101155.1 | Nidogen 1 | 28 | 7.909143 | Up | 0.00449 | other |
| *TMEM8A* | NM_001098907.1 | Transmembrane protein 8A | 25 | 4.36431 | Up | 0.0343189 | other |
| *KANK2* | NM_001076531.1 | KN motif and ankyrin repeat domains 2 | 7 | 4.302433 | Up | 0.0399683 | transcription regulator |
| *ADAM19* | NM_001075475.2 | ADAM metallopeptidase domain 19 | 7 | 3.206549 | Up | 0.0027929 | peptidase |
| *LOC615263* | NM_001103298.1 | Uncharacterized LOC615263 | 2 | 2.98966 | Down | 0.0388899 | NA |
| *REV3L* | NM_001206172.1 | REV3-like, polymerase (DNA directed), zeta, catalytic subunit | 9 | 2.219812 | Down | 0.022835 | enzyme |

**Table S5.** Differentially expressed genes from the comparison of SCNT blastocysts derived from depleted cells and nondepleted cells cultured in the absence of TSA (n = 14). Fold changes are relative to SCNT blastocysts derived nondepleted cells cultured in the absence of TSA.

| **Gene Symbol** | **Gene ID** | **Gene name** | **Chr** | **Fold change** | **Regulation** | **FDR** | **Type** |
| --- | --- | --- | --- | --- | --- | --- | --- |
| *C15H11orf34* | NM_001113538.1 | Chromosome 15 open reading frame, human C11orf34 | 15 | 7.815559 | Up | 0.029765401 | NA |
| *LOC615263* | NM_001103298.1 | Uncharacterized LOC615263 | 2 | 3.888634 | Up | 0.001795205 | NA |
| *REV3L* | NM_001206172.1 | REV3-like, polymerase (DNA directed), zeta, catalytic subunit | 9 | 2.522063 | Up | 0.001795205 | enzyme |
| *CA4* | NM_173897.1 | Carbonic anhydrase IV | 19 | 329.08 | Down | 0.003983779 | enzyme |
| *ZADH2* | NM_001075964.1 | Zinc binding alcohol dehydrogenase domain containing 2 | 24 | 78.20581 | Down | 0.011132627 | enzyme |
| *CLDN8* | NM_001098096.1 | Claudin 8 | 1 | 12.78876 | Down | 0.003983779 | other |
| *TMEM38A* | NM_001083463.1 | Transmembrane protein 38A | 7 | 7.740737 | Down | 0.011132627 | ion channel |
| *C10H5orf13* | NM_001105045.1 | Chromosome 10 open reading frame, human C5orf13 | 10 | 5.096216 | Down | 0.009116463 | NA |
| *USP3* | NM_001102123.1 | Ubiquitin specific peptidase 3 | 10 | 3.985756 | Down | 0.003983779 | other |
| *HERC3* | NM_001083663.1 | HECT and RLD domain containing E3 ubiquitin protein ligase 3 | 6 | 3.596033 | Down | 0.003983779 | enzyme |
| *FKBP9* | NM_001046372.2 | FK506 binding protein 9 | 4 | 3.083186 | Down | 0.002816989 | enzyme |
| *DCXR* | NM_001075891.1 | Dicarbonyl/L-xylulose reductase | 19 | 2.751082 | Down | 0.009630171 | enzyme |
| *SH3D21* | NM_001162534.1 | SH3 domain containing 21 | 3 | 2.420875 | Down | 0.002816989 | other |
| *FDPS* | NM_177497.2 | Farnesyl diphosphate synthase | 3 | 2.361433 | Down | 0.011132627 | enzyme |

**Table S6.** Differentially expressed genes from the comparison of SCNT blastocysts derived from depleted cells and nondepleted cells cultured in the presence of TSA (n = 35). Fold changes are relative to SCNT blastocysts derived from nondepleted cells cultured in the presence of TSA.

| **Gene Symbol** | **Gene ID** | **Gene name** | **Chr** | **Fold change** | **Regulation** | **FDR** | **Type** |
| --- | --- | --- | --- | --- | --- | --- | --- |
| *ADCY1* | NM_174229.2 | Adenylate cyclase type 1 | 4 | 263.7949 | Up | 0.0120854 | enzyme |
| *ZMAT5* | NM_001038210.2 | Zinc finger, matrin-type 5 | 17 | 62.57095 | Up | 0.0465148 | other |
| *FMR1NB* | NM_001102262.2 | Fragile X mental retardation 1 neighbor | X | 13.86671 | Up | 0.0131447 | other |
| *MAEL* | NM_001038193.2 | Maelstrom spermatogenic transposon silencer | 3 | 5.659821 | Up | 0.0498753 | other |
| *INO80E* | NM_001046544.1 | INO80 complex subunit E | 25 | 5.54002 | Up | 0.0107789 | other |
| *CLDN10* | NM_001014857.1 | Claudin 10 | 12 | 5.104958 | Up | 0.0498753 | other |
| *CHN2* | NM_001045963.2 | Chimerin 2 | 4 | 4.724665 | Up | 0.0490729 | other |
| *GLDC* | NM_001192951.1 | Glycine dehydrogenase (decarboxylating) | 8 | 3.693757 | Up | 0.0310215 | enzyme |
| *CITED2* | NM_001075819.1 | Cbp/p300-interacting transactivator, with Glu/Asp rich carboxy-terminal domain, 2 | 9 | 2.115691 | Up | 0.0498753 | transcription regulator |
| *REV3L* | NM_001206172.1 | REV3-like, polymerase (DNA directed), zeta, catalytic subunit | 9 | 2.074906 | Up | 0.0271372 | enzyme |
| *SLC6A6* | NM_174610.2 | Solute carrier family 6 (neurotransmitter transporter), member 6 | 22 | 934.6827 | Down | 0.0088673 | transporter |
| *UPP1* | NM_001098976.2 | Uridine phosphorylase 1 | 4 | 437.8524 | Down | 0.0120854 | enzyme |
| *DNASE1L3* | NM_001205724.1 | Deoxyribonuclease I-like 3 | 22 | 111.5236 | Down | 0.0055903 | enzyme |
| *TKDP1* | NM_205776.1 | Trophoblast Kunitz domain protein 1 | 13 | 54.45196 | Down | 0.0373791 | NA |
| *CA4* | NM_173897.1 | Carbonic anhydrase IV | 19 | 39.48189 | Down | 0.0498753 | enzyme |
| *NGFR* | NM_001102478.2 | Nerve growth factor receptor | 19 | 28.33934 | Down | 0.006845 | transmembrane receptor |
| *ENTPD1* | NM_174536.2 | Ectonucleoside triphosphate diphosphohydrolase 1 | 26 | 15.55199 | Down | 0.0055903 | enzyme |
| *ENPP3* | NM_001075923.2 | Ectonucleotide pyrophosphatase/phosphodiesterase 3 | 9 | 12.09189 | Down | 0.0498753 | enzyme |
| *NANOG* | NM_001025344.1 | Nanog homeobox | 5 | 11.23455 | Down | 0.0167144 | transcription regulator |
| *TPI1* | NM_001013589.3 | Triosephosphate isomerase 1 | 5 | 8.893612 | Down | 0.006845 | enzyme |
| *CD74* | NM_001034735.1 | CD74 molecule, major histocompatibility complex, class II invariant chain | 7 | 7.281397 | Down | 0.034401 | transmembrane receptor |
| *NID1* | NM_001101155.1 | Nidogen 1 | 28 | 6.338005 | Down | 0.0120854 | other |
| *SCIN* | NM_174177.2 | Scinderin | 4 | 6.096761 | Down | 0.034401 | other |
| *RNASE1* | NM_001014386.4 | Ribonuclease, RNase A family, 1 | 10 | 5.792929 | Down | 0.0120854 | enzyme |
| *TRAM2* | NM_001193150.1 | Translocation associated membrane protein 2 | 23 | 4.734895 | Down | 0.006845 | other |
| *SHISA2* | NM_001101265.1 | Shisa family member 2 | 12 | 4.688848 | Down | 0.0257855 | other |
| *KRT19* | NM_001015600.3 | Keratin 19 | 19 | 4.496904 | Down | 0.047276 | other |
| *MUC1* | NM_174115.2 | Mucin 1, cell surface associated | 3 | 4.393081 | Down | 0.0490729 | other |
| *SLC10A1* | NM_001046339.1 | Solute carrier family 10 (sodium/bile acid cotransporter), member 1 | 10 | 4.20741 | Down | 0.0168298 | transporter |
| *P2RY2* | NM_001166525.1 | Purinergic receptor P2Y, G-protein coupled, 2 | 15 | 3.94375 | Down | 0.0240448 | G-protein coupled receptor |
| *ADAM19* | NM_001075475.2 | ADAM metallopeptidase domain 19 | 7 | 3.694375 | Down | 0.0002242 | peptidase |
| *APOA1* | NM_174242.3 | Apolipoprotein A-I | 15 | 3.25112 | Down | 0.0271372 | transporter |
| *PTGES* | NM_174443.2 | Prostaglandin E synthase | 11 | 3.077688 | Down | 0.0073097 | enzyme |
| *EXOC6B* | NM_001076416.1 | Exocyst complex component 6B | 11 | 1.996047 | Down | 0.0240448 | other |
| *LDHB* | NM_001316338.1 | Lactate dehydrogenase B | 5 | 1.990428 | Down | 0.0240448 | enzyme |

**Table S7.** Functional clustering of differentially expressed genes from SCNT blastocysts derived from depleted cells in the presence of TSA when compared with SCNT blastocysts derived from nondepleted cells cultured in the presence of TSA.

| **ID** | **Molecules in Network** | **IPA Score** | **No. of focus Molecules** | **Top Diseases and Functions** |
| --- | --- | --- | --- | --- |
| 1 | ↓**ADAM19**, **↑ADCY1**, Akt, ↓**APOA1**, ↓**CA4**, CD3, ↓**CD74**, **↑CHN2**, ↓**ENTPD1**, ERK, ERK1/2, Fibrinogen, Focal adhesion kinase, **↑GLDC**, Histone h3, Jnk, ↓**KRT19**, Lectin, Mapk, ↓**MUC1**, ↓**NANOG**, NFkB (complex), ↓**NGFR**, ↓**NID1**, NPIPA8 (includes others), NT3, ↓**P2RY2**, P38 MAPK, PLC, ↓**PTGES**, Rac, Ras, ↓**SLC10A1**, TCR, ↓**UPP1** | 43 | 17 | DNA Replication, Recombination, and Repair, Nucleic Acid Metabolism, Small Molecule Biochemistry |
| 2 | **↑CITED2**, Co2+, Crip2, CRK, DDX3X, ↓**DNASE1L3**, DNTTIP1, DNTTIP2, ↓**ENPP3**, ESR1, ESR2, GCSH, **↑INO80E**, JUN, KMT2C, ↓**LDHB**, **↑MAEL**, manganese, NFAT5, PDE4A, PDLIM1, peptidase, PLXDC1, **↑REV3L**, ↓**RNASE1**, RTCB, ↓**SCIN**, ↓**SHISA2**, ↓**SLC6A6**, TBRG1, TNF, TNFAIP8, TP53, YWHAZ, **↑ZMAT5** | 27 | 12 | Cell Cycle, Cell Death and Survival, Connective Tissue Disorders |
| 3 | ACKR3, ADAM10, ADORA1, ATP2A2, BMP4, C/ebp, CFL1,  **↑CLDN10**, COL6A1, Creb, CSNK1A1, DDIT3, DHX9, EGFR, ESRRA, ↓**EXOC6B**, EZH2, FSH, GNB2L1, ILF2, ILF3, Lh,  miR-16-5p (and other miRNAs w/seed AGCAGCA), MMP7, NRG (family), PDGF BB, PLN, Pln, PRKACA, RUNX1, TIMP3, ↓**TPI1**, ↓**TRAM2**, TUBB4B, WT1 | 7 | 4 | Organismal Injury and Abnormalities, Cardiovascular System Development and Function, Cell Morphology |
| 4 | CEBPZ, CWC22, **↑FMR1NB**, NOP56, NRDC, PITHD1, RPL26L1, RRP12 | 2 | 1 | Hereditary Disorder, Neurological Disease, Organismal Injury and Abnormalities |

IPA score = -log(P-value)

Bold type indicates that the genes are present in the RNA-seq data. Non-bold type indicates that the genes are directly associated with the genes in bold, which were obtained from the Ingenuity Knowledge Base.

**Table S8.** Differentially expressed genes from the comparison of SCNT blastocysts derived from nondepleted cells cultured in the presence of TSA and depleted cells in the absence of TSA (n = 66). Fold changes are relative to SCNT blastocysts derived from depleted cells in the absence of TSA.

| **Gene Symbol** | **Gene ID** | **Gene name** | **Chr** | **Fold change** | **Regulation** | **FDR** | **Type** |
| --- | --- | --- | --- | --- | --- | --- | --- |
| *CA4* | NM_173897.1 | Carbonic anhydrase IV | 19 | 418.4826 | Up | 0.0027348 | enzyme |
| *CDKL1* | NM_001101116.1 | Cyclin dependent kinase like 1 | 10 | 395.104 | Up | 0.00462706 | kinase |
| *LRRC3* | NM_001098884.1 | Eucine rich repeat containing 3 | 1 | 257.0515 | Up | 0.03262122 | other |
| *DNASE1L3* | NM_001205724.1 | Deoxyribonuclease I-like 3 | 22 | 91.94144 | Up | 0.00388577 | enzyme |
| *GRM1* | NM_001206004.1 | Glutamate receptor, metabotropic 1 | 9 | 78.61727 | Up | 0.04646914 | G-protein coupled receptor |
| *ZADH2* | NM_001075964.1 | Zinc binding alcohol dehydrogenase domain containing 2 | 24 | 41.44099 | Up | 0.02836622 | enzyme |
| *C2H1orf64* | NM_001205359.1 | Chromosome 2 open reading frame, human C1orf64 | 2 | 26.52983 | Up | 0.00190446 | NA |
| *ABHD1* | NM_001035094.2 | Abhydrolase domain containing 1 | 11 | 21.73763 | Up | 0.01592855 | other |
| *CAPN6* | NM_001192231.1 | Calpain 6 | X | 15.49141 | Up | 0.01902024 | peptidase |
| *ENPP3* | NM_001075923.2 | Ectonucleotide pyrophosphatase/phosphodiesterase 3 | 9 | 11.75464 | Up | 0.0358311 | enzyme |
| *CLDN8* | NM_001098096.1 | Claudin 8 | 1 | 10.64353 | Up | 0.00730456 | other |
| *ENTPD1* | NM_174536.2 | Ectonucleoside triphosphate diphosphohydrolase 1 | 26 | 8.310386 | Up | 0.02582954 | enzyme |
| *CLDN3* | NM_205801.2 | Claudin 3 | 25 | 7.236978 | Up | 0.02505149 | transmembrane receptor |
| *ACY1* | NM_001077115.2 | Aminoacylase 1 | 22 | 5.500799 | Up | 0.03196951 | peptidase |
| *SLC9A3* | NM_001192154.1 | Solute carrier family 9, subfamily A (NHE3, cation proton antiporter 3), member 3 | 20 | 4.682418 | Up | 0.00399044 | ion channel |
| *SLC16A7* | NM_001076336.2 | Solute carrier family 16 (monocarboxylate transporter), member 7 | 5 | 4.591758 | Up | 0.0358311 | transporter |
| *SHISA2* | NM_001101265.1 | Shisa family member 2 | 12 | 4.331788 | Up | 0.02722233 | other |
| *ATP8A1* | NM_174838.2 | Aminophospholipid transporter (APLT), class I, type 8A, member 1 | 6 | 3.746435 | Up | 0.01048693 | transporter |
| *P2RY2* | NM_001166525.1 | Purinergic receptor P2Y, G-protein coupled, 2 | 15 | 3.708376 | Up | 0.02573171 | G-protein coupled receptor |
| *MPV17L2* | NM_001098145.2 | MPV17 mitochondrial membrane protein-like 2 | 7 | 3.464796 | Up | 0.02113438 | other |
| *HERC3* | NM_001083663.1 | HECT and RLD domain containing E3 ubiquitin protein ligase 3 | 6 | 3.362609 | Up | 0.00462706 | enzyme |
| *USP3* | NM_001102123.1 | Ubiquitin specific peptidase 3 | 10 | 3.35607 | Up | 0.01592855 | other |
| *FAM115C* | NM_001101924.1 | Family with sequence similarity 115, member C; TRP channel-associated factor 2 | 4 | 3.092064 | Up | 0.01592855 | other |
| *KDELC1* | NM_001075685.1 | KDEL motif containing 1 | 12 | 3.085235 | Up | 0.01048693 | other |
| *SLC34A2* | NM_174661.2 | Solute carrier family 34 (type II sodium/phosphate cotransporter), member 2 | 6 | 3.037836 | Up | 0.04759897 | transporter |
| *PLA2R1* | NM_175051.1 | Phospholipase A2 receptor 1 | 2 | 2.912898 | Up | 0.02573171 | transmembrane receptor |
| *RAP2C* | NM_001075700.1 | RAP2C, member of RAS oncogene family | X | 2.888357 | Up | 0.00389895 | enzyme |
| *APOA1* | NM_174242.3 | Apolipoprotein A-I | 15 | 2.877166 | Up | 0.04758037 | transporter |
| *FKBP9* | NM_001046372.2 | FK506 binding protein 9 | 4 | 2.834009 | Up | 0.00462706 | enzyme |
| *SEC11C* | NM_001075175.2 | SEC11 homolog C, signal peptidase complex subunit | 24 | 2.64647 | Up | 0.00462706 | peptidase |
| *PRPH* | NM_001102378.1 | Peripherin | 5 | 2.427003 | Up | 0.02836622 | other |
| *COTL1* | NM_001046593.1 | Coactosin-like F-actin binding protein 1 | 18 | 2.36077 | Up | 0.03320892 | other |
| *EXOC6B* | NM_001076416.1 | Exocyst complex component 6B | 11 | 2.325931 | Up | 0.00190446 | other |
| *ADAM19* | NM_001075475.2 | ADAM metallopeptidase domain 19 | 7 | 2.323856 | Up | 0.04646914 | peptidase |
| *LRRC59* | NM_001034578.1 | Leucine rich repeat containing 59 | 19 | 2.319908 | Up | 0.0358311 | other |
| *SLC11A2* | NM_001101103.1 | Solute carrier family 11 (proton-coupled divalent metal ion transporter), member 2 | 5 | 2.071869 | Up | 0.02582954 | transporter |
| *MESDC2* | NM_001034469.1 | mesoderm development candidate 2 | 21 | 2.027434 | Up | 0.02573171 | other |
| *SNX2* | NM_001038519.1 | Sorting nexin 2 | 7 | 2.005639 | Up | 0.04646914 | transporter |
| *FUT8* | NM_177501.2 | Fucosyltransferase 8 (alpha (1,6) fucosyltransferase) | 10 | 1.902389 | Up | 0.02836622 | enzyme |
| *ADCY1* | NM_174229.2 | Adenylate cyclase type 1 | 4 | 679.3225 | Down | 0.00190446 | peptidase |
| *ETNPPL* | NM_001015605.1 | Ethanolamine-phosphate phospho-lyase | 6 | 43.67454 | Down | 0.02836622 | enzyme |
| *FMR1NB* | NM_001102262.2 | Fragile X mental retardation 1 neighbor | X | 18.72273 | Down | 0.00336912 | other |
| *ADRB2* | NM_174231.1 | Adrenoceptor beta 2 | 7 | 14.66617 | Down | 0.04646914 | G-protein coupled receptor |
| *LIMCH1* | NM_001191521.1 | LIM and calponin homology domains 1 | 6 | 10.08178 | Down | 0.02059606 | other |
| *TGFBI* | NM_001205402.1 | Transforming growth factor beta induced | 7 | 7.94732 | Down | 0.00190446 | other |
| *C15H11orf34* | NM_001113538.1 | Chromosome 15 open reading frame, human C11orf34 | 15 | 7.03298 | Down | 0.02573171 | NA |
| *ZBTB48* | NM_001192979.1 | Zinc finger and BTB domain containing 48 | 16 | 5.994104 | Down | 0.00730456 | transcription regulator |
| *INO80E* | NM_001046544.1 | INO80 complex subunit E | 25 | 5.75852 | Down | 0.00462706 | other |
| *CLDN10* | NM_001014857.1 | Claudin 10 | 12 | 5.127475 | Down | 0.03262122 | other |
| *JAKMIP2* | NM_001075897.2 | Janus kinase and microtubule interacting protein 2 | 7 | 4.472857 | Down | 0.01592855 | other |
| *DYRK3* | NM_001100298.1 | Dual specificity tyrosine-(Y)-phosphorylation regulated kinase 3 | 16 | 4.347433 | Down | 0.01592855 | kinase |
| *KLK6* | NM_001101140.2 | Kallikrein related peptidase 6 | 18 | 4.195303 | Down | 0.00335575 | peptidase |
| *HRH1* | NM_174083.4 | Histamine receptor H1 | 22 | 4.19463 | Down | 0.00864399 | G-protein coupled receptor |
| *MDM2* | NM_001099107.1 | MDM2 proto-oncogene, E3 ubiquitin protein ligase | 5 | 3.36033 | Down | 0.02573171 | transcription regulator |
| *STXBP1* | NM_174619.3 | syntaxin binding protein 1 | 11 | 2.896631 | Down | 0.01249247 | transporter |
| *NDRG1* | NM_001035009.2 | N-myc downstream regulated 1 | 14 | 2.798522 | Down | 0.02113438 | kinase |
| *PTK7* | NM_001192965.2 | Protein tyrosine kinase 7 | 23 | 2.677134 | Down | 0.0358311 | kinase |
| *SPARC* | NM_174464.2 | Secreted protein, acidic, cysteine-rich (osteonectin) | 7 | 2.623199 | Down | 0.00975049 | other |
| *TRIM32* | NM_001075824.2 | Tripartite motif containing 32 | 8 | 2.570942 | Down | 0.04646914 | transcription regulator |
| *TIMP2* | NM_174472.4 | TIMP metallopeptidase inhibitor 2 | 19 | 2.56774 | Down | 0.0184842 | other |
| *ESRP2* | NM_001205685.1 | Epithelial splicing regulatory protein 2 | 18 | 2.403106 | Down | 0.0261967 | other |
| *REV3L* | NM_001206172.1 | REV3-like, polymerase (DNA directed), zeta, catalytic subunit | 9 | 2.357427 | Down | 0.00335575 | enzyme |
| *FLNC* | NM_001206990.1 | Filamin C, gamma | 4 | 2.258898 | Down | 0.01060247 | other |
| *ZNF800* | NM_001205671.1 | Zinc finger protein 800 | 4 | 2.247516 | Down | 0.03437807 | other |
| *SMAD1* | NM_001076223.2 | SMAD family member 1 | 17 | 2.217787 | Down | 0.02836622 | transcription regulator |
| *C18H16orf70* | NM_001105502.1 | Chromosome 18 open reading frame, human C16orf70 | 18 | 2.020383 | Down | 0.04646914 | NA |

**Table S9.** Functional clustering of differentially expressed genes from SCNT blastocysts derived from nondepleted cells cultured in the presence of TSA when compared with SCNT blastocysts derived from depleted cells in the absence of TSA.

| **ID** | **Molecules in Network** | **IPA Score** | **No. of focus Molecules** | **Top Diseases and Functions** |
| --- | --- | --- | --- | --- |
| 1 | ADCY, ↓**ADCY1**, ↓**ADRB2**, Alp, **↑CA4**, cldn, **↑CLDN3**, **↑CLDN8**, ↓**CLDN10**, Collagen type I, Collagen(s), ↓**DYRK3**, **↑ENTPD1**, ERK, ERK1/2, estrogen receptor, ↓**FLNC**, FSH, **↑FUT8**,  G protein beta gamma, **↑GRM1**, Lh, **↑LRRC59**, ↓**NDRG1**, **↑P2RY2**, Pka, PLC, **↑RAP2C**, **↑SLC34A2**, **↑SLC9A3**, ↓**SMAD1**, ↓**SPARC**, Tgf beta, ↓**TGFBI**, ↓**TIMP2** | 49 | 21 | Cellular Function and Maintenance, Molecular Transport, Cell-To-Cell Signaling and Interaction |
| 2 | 26s Proteasome, **↑ADAM19**, ADRA2C, Akt, **↑APOA1**,  Beta Arrestin, CD3, CYTIP, Focal adhesion kinase, Gpcr, GPRC5B, hemoglobin, **↑HERC3**, Histone h3, ↓**HRH1**, Insulin, Jnk, ↓**MDM2**, NFkB (complex), P38 MAPK, Pkc(s), ↓**PLA2R1**, ↓**PRPH**, ↓**PTK7**, Rac, **↑SHISA2**, **↑SLC11A2**, SLC52A2, SLC6A2, ↓**STXBP1**, ↓**TRIM32**, Ubiquitin, **↑USP3**, Vegf, ↓**ZBTB48** | 28 | 14 | Psychological Disorders, Organismal Injury and Abnormalities, Skeletal and Muscular Disorders |
| 3 | **↑ATP8A1**, BDNF, Ca2+, **↑CDKL1**, CLASP1, **↑DNASE1L3**, EDIL3, EGFR, ELAVL1, **↑ENPP3**, **↑EXOC6B**, **↑FKBP9**, GUSB, HOMER2, HSD17B12, HTR2A,IL13, ↓**JAKMIP2**, melanin, mGluR, mGLUR Group I, NHLH1, PLA2G2D, PSMB5, PTGDR2, RAB18, SIRT1, **↑SLC16A7**, **↑SNX2**, TM9SF3, TNF, TRPC1, TRPC3, **↑ZADH2**, ↓**ZNF800** | 21 | 11 | Cell Signaling, Small Molecule Biochemistry, Vitamin and Mineral Metabolism |
| 4 | **↑ACY1**, ALDH4A1, APP, **↑CAPN6**, CCND1, CEBPZ, COL4A6, CPEB1, CWC22, EED, ↓**ETNPPL**, ↓**FMR1NB**, HNF4A, HOMER2, JKAMP, **↑KDELC1**, ↓**KLK6**, ↓**LIMCH1**, **↑LRRC3**, **↑MESDC2**, NRDC, OTUD6B, Ppp1cc, RAB18, ↓**REV3L**, RPL26L1, RRP12, **↑SEC11C**, STOM, SUCLG1, SYNJ2BP, TACO1, TMEM33, TP53, USP48 | 20 | 11 | DNA Replication, Recombination, and Repair, Cancer, Developmental Disorder |
| 5 | ALDH2, ALOX5AP, BRD7, **↑COTL1**, ELF4, **↑ENTPD1**, ↓**ESRP2**, FGFR2, FKBP5, FOXP3, GEM, INO80, ↓**INO80E**, ITPR1, KDM6B, KPNA3, MAP4, MCRS1, MOG, MXD1, NRP1, PRPF6, PSMC1, S100A6, SEMA3B, SERPINH1, SFTPA1, SMARCA4, **↑TCAF2**, TGFB1, TNFAIP8, TNFRSF14, TUBA3C/TUBA3D, UBD, USO1 | 8 | 5 | Lipid Metabolism, Small Molecule Biochemistry, Cellular Movement |
| 6 | **↑ABHD1**, CCDC155 | 2 | 1 | Cell Cycle, DNA Replication, Recombination, and Repair, Cellular Development |

IPA score = -log(P-value)

Bold type indicates that the genes are present in the RNA-seq data. Non-bold type indicates that the genes are directly associated with the genes in bold, which were obtained from the Ingenuity Knowledge Base.
